# Ultrafine mapping of chromosome conformation at hundred basepair resolution reveals regulatory genome architecture

**DOI:** 10.1073/pnas.2313285120

**Published:** 2023-11-03

**Authors:** Yizhou Zhu, Michael G. Rosenfeld, Yousin Suh

**Affiliations:** ^a^Department of Obstetrics and Gynecology, Columbia University, New York, NY 10032; ^b^Department of Medicine, University of California San Diego, La Jolla, CA 92093; ^c^Department of Genetics and Development, Columbia University, New York, NY 10032

**Keywords:** chromatin conformation, Hi-C, gene regulation, molecular genetics

## Abstract

The resolution limit of chromatin conformation capture methodologies (3Cs) has restrained their application in detection of fine-level chromatin structure mediated by cis-regulatory elements (CREs). Here, we report two 3C-derived methods, Tri-4C and Tri-HiC, which utilize multirestriction enzyme digestions for ultrafine mapping of targeted and genome-wide chromatin interaction, respectively, at up to one hundred basepair resolution. Tri-4C identified CRE loop interaction networks and quantitatively revealed their alterations underlying dynamic gene control. Tri-HiC uncovered global fine-gauge regulatory interaction networks, identifying >20-fold more enhancer:promoter (E:P) loops than in situ Hi-C. In addition to vastly improved identification of subkilobase-sized E:P loops, Tri-HiC also uncovered interaction stripes and contact domain insulation from promoters and enhancers, revealing their loop extrusion behaviors resembling the topologically associating domain boundaries. Tri-4C and Tri-HiC provide robust approaches to achieve the high-resolution interactome maps required for characterizing fine-gauge regulatory chromatin interactions in analysis of development, homeostasis, and disease.

## Significance

In this research, we developed Tri-4C and Tri-HiC, innovative approaches that significantly improve the resolution of chromosome conformation mapping. These methods effectively overcome the limitations of traditional chromatin conformation capture techniques, enabling robust detection of intricate chromatin structures formed at cis-regulatory elements (CREs) in sub-TAD (topologically associating domain) levels. By unveiling the fine-gauge architecture of the regulatory genome with extensive detail, this work offers an insight for how distal CREs physically communicate and drive long-range gene regulation.

Transcriptional regulation in the mammalian genome involves interplay between promoters at the transcription start sites and distal cis-regulatory elements (CREs), particularly enhancers. While an ever-increasing number of methodologies have been established to characterize the location and activity of distal CREs, fewer are available for assessing their physical interactions (1, 2). The chromosome conformation capture (3C) and its derived assays revolutionized the understanding of the chromatin architecture, revealing folding structures known as topologically associating domains (TADs) bound by looped boundaries harboring orientation-specific CTCF pairs and cohesins (3). However, recent studies suggest a somewhat limited impact of TAD and boundary loops on gene regulation than initially proposed (4, 5). On the other hand, interactions between CREs have been implicated to regulate gene expression by multiple approaches, including histone quantitative trait loci (6), enrichment-based loop detection methods such as proximity ligation-assisted chromatin immunoprecipitation sequencing (PLAC-seq) and HiChIP (7–9), and forced chromatin looping (10). Revelation of the fine layer structure at sub-TAD level is thus critical to understand gene regulation from the perspective of chromatin conformation. However, such microstructures can only be vaguely interrogated by current 3C methods such as 4C-seq and Hi-C (3, 11) due to their kilobase-range analytical resolutions much below the CRE mapping techniques.

Despite numerous branches of derivatives of 3C being developed, most of them share a conserved principle to map proximity-ligated genome fragments generated by restriction digestion (2). Consequently, the detection of contact frequencies is limited at the restriction sites, resulting in a theoretical resolution cap for the methods. Although a 4-bp cutter restriction enzyme (RE) typically used in the current methods yields on average of 256 bp fragments, the static distribution of restriction sites on the genome results in a significant portion of loci being consistently underdigested with >1-kb gap intervals (*SI Appendix*, Fig. S1*A*). It is likely that small CREs (e.g., 200 to 300 bp core size for enhancers 12, 13) within these gaps are insufficiently tagged, raising concerns whether their interaction signals can be robustly detected. While alternative digestion approaches, including use of MNase and DNase (14, 15), have been proposed to address such concern, these enzymes can introduce additional bias due to their differential cutting efficiency at nucleosome-free CREs and the rest of the genome. Therefore, novel high-resolution methods are required for comprehensive interrogation of cis-regulatory interactions in the genome.

## Results

### Tri-4C Refines Genome Fragmentation by Triple 4-Cutter Digestion.

**Fig. 1.**
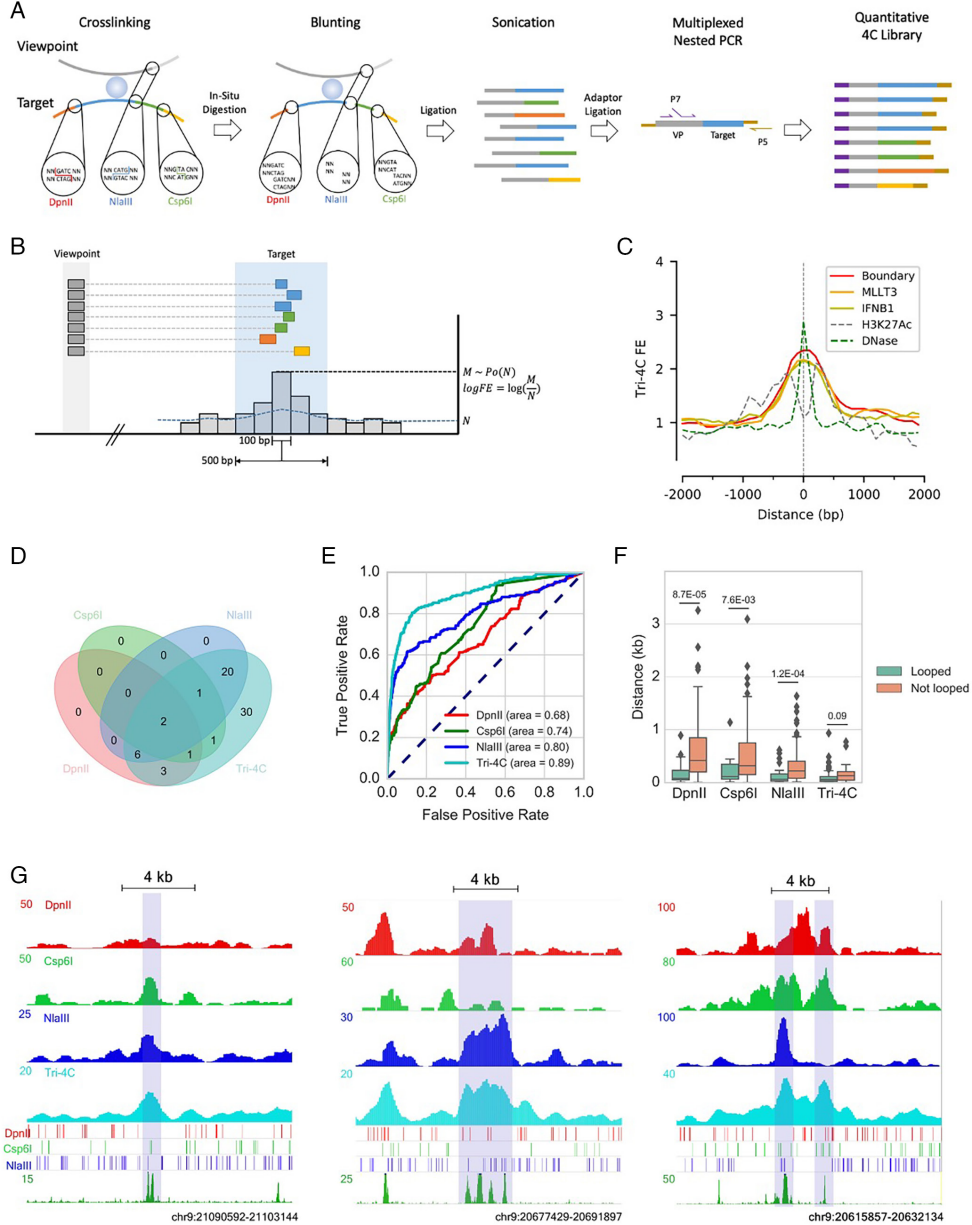
Tri-4C robustly and unbiasedly identifies CRLs. (*A*) Schematics of Tri-4C library construction. (*B*) Tri-4C loop calling algorithm. Each 100 bp sliding bin collects read count (*M*) from neighboring 500 bp intervals. Significant loops and their loop strengths are determined by Poisson statistics of *M* against expected read count *N* calculated from the total read counts in the 5 to 50 kb local background. (*C*) Center Plot of Tri-4C signals at all DHS-marked CREs in the 9p21 *IFNB1* TAD. (*D*) Venn diagram of reproducible CRLs (N = 2) called for the Boundary viewpoint using Tri-4C and UMI-4C digested by three different REs (*E*) ROC analysis using loop signals [−log(p)] for each 100 bp bin steps from Boundary as predictors of intra-TAD DHS peaks. (*F*) Boxplot for distance between intra-TAD DHS peak (N = 85) center and the closest restriction site, separated by whether the peak is called to loop with any of the three viewpoints. (*G*) Correlation between raw signals of Tri-4C and UMI-4C and neighboring restriction site patterns at three CRE regions looped with Boundary. Statistic p values were calculated by the *U* test. The *y* axis of 4C methods denotes read count per 10,000 uniquely mapped reads.

To overcome the limitations of current methods to comprehensively detect CRE loops, we developed Tri-4C, a targeted chromatin conformation capture (4C) method. In Tri-4C, distal chromatin interactions are probed by in situ digestion of genomic DNA using three 4-bp cutter REs, DpnII (MboI), Csp6I (CviQI), and NlaIII (Fig. 1*A*). In silico analysis of the human genome showed that the fragment size of Tri-4C is 1.9- to 5.2-fold shorter than that of single 4-bp cutters (*SI Appendix*, Fig. S1*A*). The sticky ends are then blunted, allowing free religation of cutting sites generated by three different REs, which dramatically increases ligation complexity. After sonication, the enrichment of contacts at the target viewpoint is achieved by two rounds of nested PCR using two sequential primers in the vicinity of the cutting site. The chromatin contacts are then identified through paired-end sequencing. Similar to UMI-4C (11), the sonication ends are utilized as unique molecular identifiers (UMI) to generate a PCR bias-free quantitative interaction map. The Tri-4C protocol can be multiplexed, and the procedure can be completed in 3 to 4 d.

To test the performance of Tri-4C, we applied the method to examine the 9p21 interferon B1 (*IFNB1*) TAD, which harbors 85 putative CREs marked by DNase I hypersensitive sites (DHSs) in IMR90 cells (16). In situ Hi-C reveals only 8 loops within the TAD that are solely between binding sites of CCCTC-binding factors (CTCF), suggesting that the cis-regulatory interactions within the contact domain remain largely elusive (*SI Appendix*, Fig. S2). We generated the Tri-4C distal interaction profiles on three viewpoints, two promoters (*MLLT3* and *IFNB1*) and a sub-TAD boundary (Boundary) showing strong CTCF/cohesin binding (Dataset S1 and *SI Appendix*, Fig. S2). To compare with existing techniques, we performed UMI-4Cs (with minor modifications—see *Materials and Methods*) digested individually by DpnII, Csp6I, or NlaIII in parallel on the same viewpoints (11). To adapt to the multiple ligation ends generated by Tri-4C, we developed a modified pipeline that retained the UMI-recognition property of UMI-4C to remove PCR duplicates, thereby generating quantitative interaction profiles (*Materials and Methods*). We found that using the same cell number input, Tri-4C generated on average 5.3-fold more unique contacts than UMI-4C (Dataset S2 and *SI Appendix*, Fig. S1*B*), suggesting that the detectability of distal interaction proportionally increased with digestion frequency. Consistently, the reproducibility of Tri-4C was significantly higher than UMI-4C, especially at subkilobase resolution (*SI Appendix*, Fig. S1*C*).

### Tri-4C Comprehensively Identifies Cis-Regulatory Loops at Hundred Basepair Resolution.

In order to differentiate interaction loops from the local background interactions that occur with high frequency within TADs, we developed an algorithm resembling MACS (17) to identify loop sites with overrepresented interaction read counts. Since 97% of fragments generated by the triple digestion are smaller than 500 bp, we binned reads into 500 bp windows in 100 bp sliding steps, a resolution comparable to the size CREs, and quantified their enrichment against a local background within a 5 to 50 kb dynamic range (Fig. 1*B* and *SI Appendix*, Fig. S1*A*). We applied the algorithm to Tri-4C, yielding 233, 138, and 21 reproducible intra-TAD loops, respectively, for the *MLLT3*, Boundary, and *IFNB1* viewpoints. These loops significantly overlapped with a total of 70 CREs marked by DHS, 37 of which were also marked by H3K27Ac (*SI Appendix*, Fig. S3), which achieved a 4.6-fold increase compared to UMI-4Cs (Fig. 1*D* and *SI Appendix*, Fig. S6*A*). The loop score of Tri-4C more accurately predicted the positions of DHS-marked CREs and H3K27Ac-marked enhancers than UMI-4C, suggesting that its higher detection sensitivity of cis-regulaotry loops (CRLs) was not compromised by specificity (Fig. 1*E* and *SI Appendix*, Fig. S6 *B* and *C*). We also examined the mappability, GC content, and restriction site density around the identified loops, finding that loop calling was not significantly affected by these considerations (*SI Appendix*, Fig. S3 *C*–*E*).

The 500 bp resolution (bin size) we chose to perform loop calling for Tri-4C was significantly greater than those used by UMI-4C (3 to 5 kb) or Hi-C/HiChIP (5 kb). To test the impact of higher resolution on CRL detection, we reanalyzed the Tri-4C data with a larger bin size (3000 bp), comparable to previous methods (3, 11, 18). At 3 kb resolution, Tri-4C identified on average 35% of the CRLs found at 500 bp resolution (*SI Appendix*, Fig. S4), with lower signal-to-noise ratios at the overlapping loops, and produced merged loop signals between closely located CREs. Consistently, the 500 bp resolution analysis revealed that CRLs were less than 1 kb long, with the pinnacle precisely aligning with DHS peaks (Fig. 1*C*). Hence, subkilobase resolution mapping was essential to prevent excess convolution with background, robustly identifying CRLs.

We compared the Tri-4C loop caller with UMI-4C and 1D adaptation of in situ Hi-C algorithms, both of which estimate background interactions by using distance modeling based on global interaction profiling (*Materials and Methods*). Receiver operating characteristic (ROC) analysis at 100 bp resolution showed that Tri-4C loops were a strong predictor of DHS-marked CREs regardless of the algorithm used, while loop scores determined by the Tri-4C caller showed the highest accuracy (*SI Appendix*, Fig. S5*A*). Furthermore, the CRL strengths (fold-enrichment against background) determined by the Tri-4C algorithm were distance-independent and strongly correlated between viewpoints (r = 0.82 between Boundary and *MLLT3*). The correlations obtained by the Hi-C and UMI-4C algorithms were less significant (r = 0.48 and 0.29, respectively), probably because of their tendency to overcorrect for the distance (*SI Appendix*, Fig. S5 *B*–*D*).

### Digestion at Close Vicinity is Essential for Detecting Cis-Regulatory Loops.

The UMI-4C profiles generated by three different 4-bp cutters revealed poorly overlapped subsets of the CRLs identified by Tri-4C (intersection over union < 0.2) (*SI Appendix*, Fig. S6*A*), suggesting that the detection of CRLs by UMI-4C was selective and enzyme-dependent. We case-studied loops that were identified by at least one but not all three UMI-4C profiles, and found that profiles failed to detect the loop invariantly lacked restriction sites nearby the CRE (Fig. 1*G*). Consistently, the overall distances between the looped CRE and its nearest restriction site were significantly higher than the unlooped for all UMI-4Cs (Fig. 1*F*). Such correlation was not found in Tri-4C, suggesting that its ultrafine digestion of the genome was necessary and sufficient to address the restriction site-dependent loop detection bias. Quantification of loop strength exhibited a negative correlation between the distance between the CRE and the restriction site for UMI-4Cs compared to Tri-4C, suggesting that insufficient genome digestion by single RE reduced loop detection sensitivity and dampened their peak strengths (*SI Appendix*, Fig. S7).

### Tri-4C Loop Profile Is Indicative of CRE Activities.

We further investigated Tri-4C-identified loop sites that did not overlap with enhancer marks, including histone modifications and DHS. We found them partially overlapped with ENCODE ChIP-seq signals of transcription factors, suggesting their regulatory potential (*SI Appendix*, Fig. S8*A*). To determine possible regulatory function of these loops, we used CRISPR/Cas9 to delete ~1 kb regions of 4 sites that looped with the *MLLT3* promoter but were devoid of enhancer marks (*SI Appendix*, Fig. S8 *B* and *C* and Dataset S3). We found that deletion of two of the sites significantly down-regulated *MLLT3* expression, indicating that these were bona fide enhancers (*SI Appendix*, Fig. S8*D*). Of note, these functional enhancer loops had escaped detection by DpnII UMI-4C.

To quantitatively analyze the CRLs called by Tri-4C, we compared the loop strength (i.e., log fold enrichment against local background) with the DHS fold enrichment for all CREs in the locus, finding that they were significantly correlated (*SI Appendix*, Fig. S9*A*). Motif analysis indicated that CREs harboring the CTCF motif formed significantly stronger loops with all three viewpoints (*SI Appendix*, Fig. S9*B*), consistent with the role of CTCF in mediating chromatin interactions (3, 19). In contrast, this correlation was not revealed when analyzed using UMI-4C.

To test whether Tri-4C can reveal the CRL networks underlying dynamic gene control, we induced robust expression of *IFNB1* through activation of well-defined antiviral signaling and performed Tri-4C on all three viewpoints (20). The induction of *IFNB1* caused its promoter to interact more frequently with the majority of CREs in the locus (*SI Appendix*, Fig. S10 *A*–*C*). However, many of these gains were not significant against the similarly increased local background, and after normalization only 13 CREs showed induced looping with *IFNB1* (*SI Appendix*, Fig. S10 *D* and *E*). The alterations in loop strength with the *IFNB1* promoter significantly correlated with those from the *MLLT3* and Boundary viewpoints, as well as the CRE activities indicated by the ATAC-seq peak strengths (*SI Appendix*, Fig. S10 *F* and *G*). The CREs gaining loop strength upon induction were enriched with the motifs of IRF family members, which are key regulators for *IFNB1* activation (*SI Appendix*, Fig. S10*H*) (21). These results indicated that Tri-4C provides the sensitivity and specificity capable of revealing quantitative loop alterations underlying the activities of CREs in the CRL networks.

### Allele-Specific (AS) Tri-4C Quantitatively Reveals SNP-Associated Loop Alterations.

To test whether Tri-4C could differentiate the allelic impact of regulatory variants on CRL networks, we applied Tri-4C to examine the 9p21.3 locus. This locus harbors multiple coronary artery disease (CAD) risk variants, including two functional variants reported to abrogate the function of an enhancer (*ECAD9*) by disrupting TEAD3 and STAT1 binding, thereby misregulating the expression of the target genes, *CDKN2A/B* (22–24). Using vascular smooth muscle cells (VSMC) derived from a human embryonic stem cell line (H7) that is heterozygous for the risk variants, we performed AS Tri-4C on ECAD9 (*SI Appendix*, Fig. S11*A*) (23). The AS-Tri-4C profile showed highly cis-specific interaction (>99%), revealing looping of ECAD9 with 25 ATAC-seq-marked CREs in the locus, including both *CDKN2A* and *CDKN2B* promoters (*SI Appendix*, Fig. S11 *B* and *C*). Among the looped CREs, 10 showed differential loop strength between alleles, and in all cases, loops on the nonrisk alleles were significantly stronger than those of the risk alleles. The stronger loop activity of *ECAD9* on the nonrisk allele was consistent with its higher accessibility indicated by ATAC-qPCR (*SI Appendix*, Fig. S11*D*) (25). Last, we found that stronger loops were formed between *ECAD9* and CREs harboring TEAD3, STAT1, or SMAD family motifs (*SI Appendix*, Fig. S11*E*), consistent with the roles of these factors in regulating *CDKN2A/B*, which are diminished by the CAD risk variants (22, 23, 26).

### Tri-HiC Maps Global Chromatin Interaction at a Hundred Basepair Resolution with Low Cell Input.

**Fig. 2.**
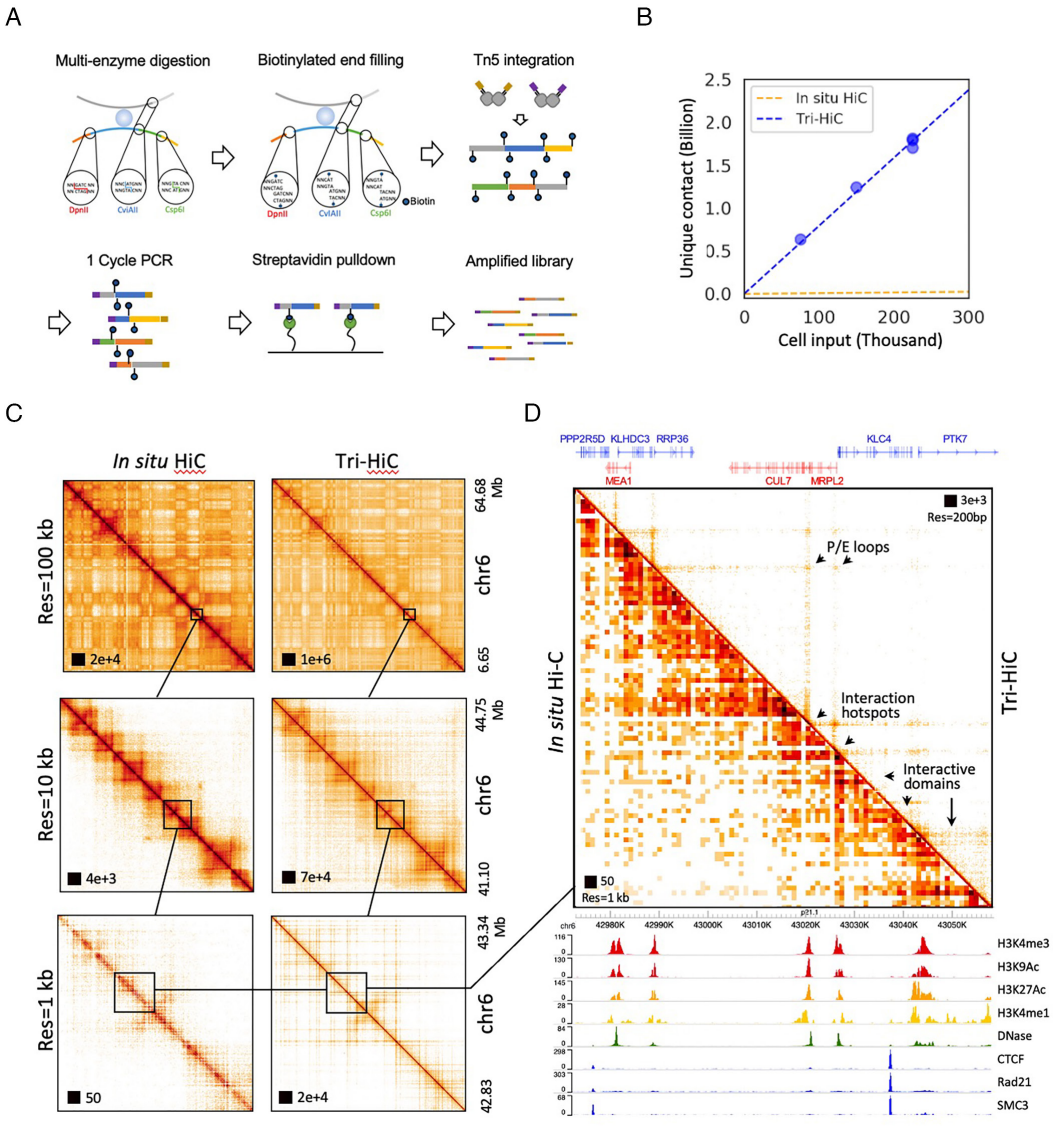
(*A*) Schematics of Tri-HiC library construction. (*B*) Yield curve of Tri-HiC (N = 5) in comparison with yield of in situ Hi-C for IMR90 (N = 7) reported by Rao et al. (3), assuming that each in situ Hi-C library was constructed with 2 million cells as minimum suggested by authors. (*C*) An example comparison of interaction maps of a locus on chromosome 6 between Tri-HiC and in situ Hi-C at multiple resolutions. (*D*) A 90 kb zoomed-in region from *C* with gene and epigenetic annotations. Arrows highlight microstructural features uniquely revealed by Tri-HiC, including interaction hotspots (anchors for nonspecific interaction stripes), promoter-enhancer (P/E) loops, and interaction microdomains.

Since Tri-4C substantially improves the resolution and CRL detection, we next applied the analogous multienzyme approach to develop Tri-HiC for global distal chromatin contact mapping at a hundred basepair resolution. Similar to Tri-4C, the Tri-HiC method utilizes three REs to increase genome digestion with the only difference being replacing NlaIII with its isoschizomer, CviAII, to generate 5′ AT overhangs for the biotin labeling (Fig. 2*A*). We tested this enzyme combination with Tri-4C on the Boundary and *MLLT3* viewpoints and found the alternatively digested interaction profiles were highly consistent with Tri-4C using NlaIIl digestion (r = 0.94) (*SI Appendix*, Fig. S12). To maximize the yield and sequencing efficiency of Tri-HiC, we used Tn5 tagmentation (Illumina), instead of sonication and TA ligation employed in in situ Hi-C, to generate short fragment libraries. Notably, this modification also removed unligated ends due to the insertion nature of transposons. Furthermore, the Tn5 tagmentation step permitted a PCR cycle prior to streptavidin pulldown, which separated the two biotin-labeled strands to increase the capture efficiency for the contacts.

We generated Tri-HiC libraries for 5 biological replicates for IMR90 with a total sequencing coverage of 13 billion raw read counts (Dataset S4 and *Materials and Methods*). With 80,000 to 240,000 cell inputs for each replicate, we obtained ~0.6 to 1.8 billion unique contacts, scaling linearly with the input (Fig. 2*B*), and 7.2 billion unique contacts in total for all replicates combined. This yield efficiency is about 70-fold more efficient than in situ Hi-C, which typically obtains a few hundred million contacts from 2 to 5 million cells (3). Reproducibility tests indicated that Tri-HiC maintained high scores (Pearson R > 0.85) across replicates at a range of resolution up to 500 bp (*SI Appendix*, Fig. S13*A*).

Analysis of contact frequency against genomic distance indicated their overall log-linear negative correlation (*SI Appendix*, Fig. S13*B*). We observed an up to 10-fold overrepresentation of out–out contacts in the 100 bp to 10 kb range, compared to the in–out and out–in pattern, which could indicate that circularized ligation between two ends of the fragment is favored in short distance. Interestingly, the out–out frequency displayed a distinct waving pattern at subkilobase range with peaks at 200, 600, and 1,000 bp, suggesting stable short-range structures associated with nucleosome organizations.

We used Hi-C pipelines Juicer (27) and Distiller (28) to map the Tri-HiC libraries with resolution settings up to 100 bp. To evaluate the results, we compared the interaction profiles with the in situ Hi-C data for IMR90 (1.1 billion contacts) (3). At lower resolutions (10 to 100 kb), Tri-HiC recapitulated the macrostructures such as chromatin compartments and TADs revealed by in situ Hi-C (Fig. 2*C*). At 1 kb and subkilobase resolutions; however, Tri-HiC uniquely identified multiple microstructures which were not visible in in situ Hi-C, including the nonspecific interaction stripes extended from distal interaction hotspots, the sub-TAD loops, and interactive microdomains with sizes as small as a few kb (Fig. 2*D*). Contrary to the CTCF/cohesin-centric chromatin architecture revealed by in situ Hi-C, these microstructures were aligned to active CREs such as promoters and enhancers with no detectable CTCF binding, revealing their essential roles in organizing the sub-TAD chromatin architecture. Notably, these structures are consistent with the fine-gauge interactions recently reported by Micro-C, a high-resolution Hi-C technique utilizing MNase as an alternative approach to overcome the restriction digestion size limit (15). However, compared to Micro-C, which reported library constructions using up to 10^7^ cells (15), Tri-HiC revealed these structures with substantially lower cell input (Fig. 2*B*).

To confirm that the identification of the CRE-associated structures is not due to our ultrahigh sequencing depth, we specifically compared in situ Hi-C with a Tri-HiC replicate with similar coverage (1.2 billion contacts). In all loci examined, we found that despite some resolution compromise, the low-depth profile recapitulated the structural features revealed by the pooled Tri-HiC library (*SI Appendix*, Fig. S14). We also noticed that the same 1 kb resolution was sufficient to identify the CRE-associated structures by Tri-HiC but not in situ Hi-C, suggesting that the improvement was not due to the arbitrarily defined resolution bin size but rather the increased detection of interactions, analogous to the demonstrated advantages of Tri-4C.

### Tri-HiC Robustly Identifies Cis-Regulatory Loops Genome-Wide.

**Fig. 3.**
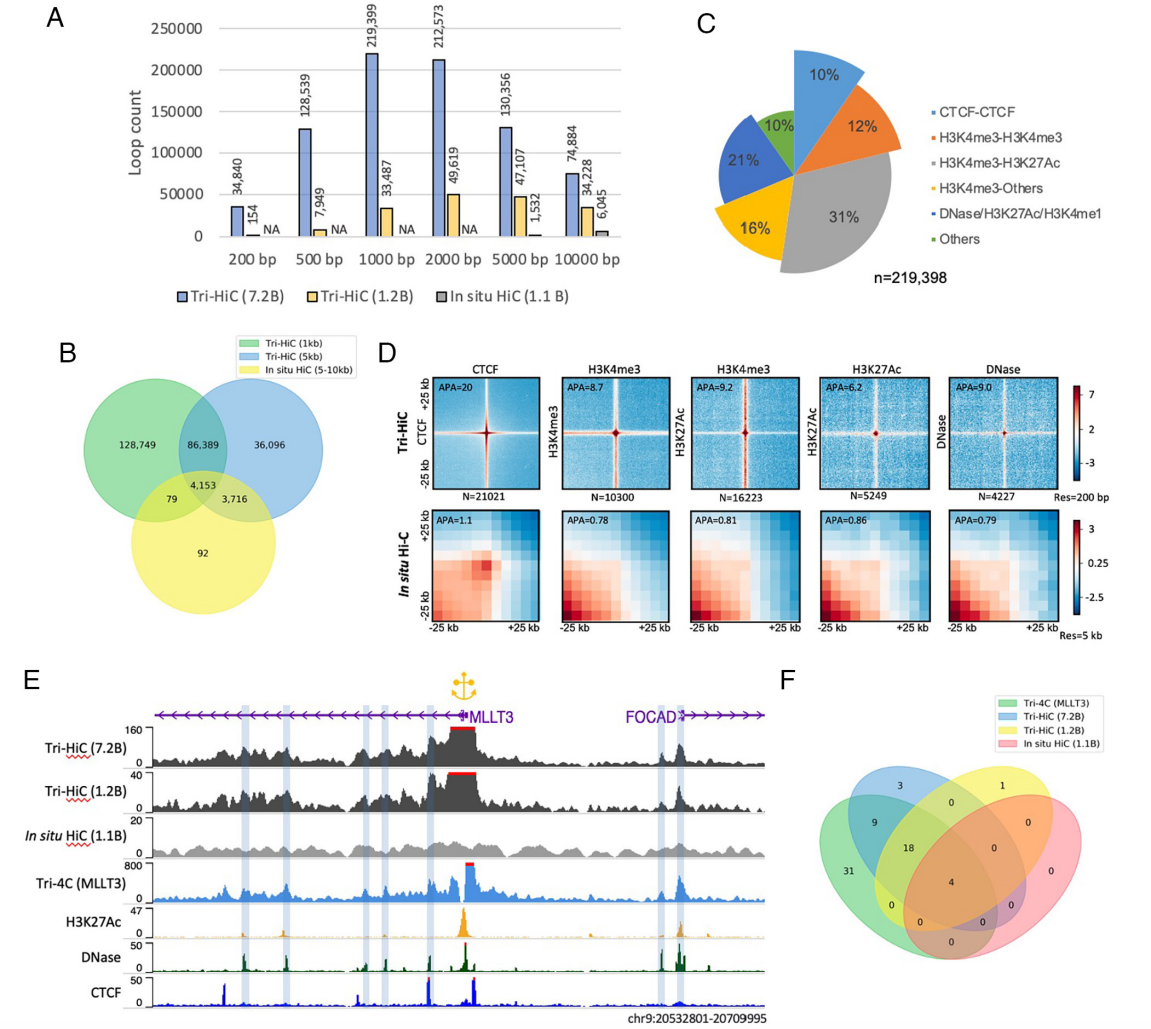
Identification of cis-regulatory loops by Tri-HiC. (*A*) Number of loops called by HICCUPS for Tri-HiC all (7.2 billion contacts), replicated #2 (1.2 billion contacts), and in situ Hi-C (1.1 billion contacts) at different resolutions for IMR90 cells. (*B*) Venn diagram indicating the overlap statistics of loops identified by Tri-HiC at 1 kb and 5 kb resolutions and in situ Hi-C. (*C*) Annotation of loops identified at 1 kb resolution. Loop annotations with multiple categories are classified to the category with the largest bar size. (*D*) Z-score transformed aggregated peak analysis (APA) of loops identified by Tri-HiC at 1 kb resolution sorted by annotations. The aggregated heatmaps are visualized in 200 bp resolution for Tri-HiC and 5 kb for in situ Hi-C. Loops annotated into multiple categories are included only once into the leftmost figure. (*E*) Comparison virtual 4Cs derived from Tri-HiC and in situ Hi-C with Tri-4C for *MLLT3*. (*F*) Overlaps of *MLLT3* intra-TAD DNase loops identified by different methods.

To identify significant loop interactions, we applied the HiCCUPS (27) algorithm to Tri-HiC at multiple resolutions. A highest number of 219,399 loops was identified from the 7.2 B pooled library at 2 kb resolution (Fig. 3*A*). These number was 27.2-fold and 7.42-fold higher than 8,040 total loop counts reported by in situ Hi-C (3) and 29,548 reported by Micro-C (15), respectively. Notably, at 1 to 2 kb range, in situ Hi-C failed to reveal any loops, suggesting finer digestion by Tri-HiC is essential for high-resolution loop calling. Overlapping the loop profiles between the two assays, we found that Tri-HiC reproduced 98% of in situ Hi-C loops at 5 kb resolution (Fig. 3*B*). Interestingly, 3,716 in situ Hi-C loops were found only at 5 kb but not at 1 kb resolution, which could indicate limitations of loop detection power at high resolution, possibly due to coverage insufficiency.

We next annotated the 1 kb loops identified by Tri-HiC. The highest fraction of loops (31%) were found between promoters (H3K4me3) and active enhancers (H3K27Ac); by contrast, only 10% of the loops were bound by CTCF sites (Fig. 3*C*). This composition is distinct from in situ Hi-C, for which the majority of loops (58%) were identified between CTCFs (*SI Appendix*, Fig. S15*A*). The vast majority (90%) of Tri-HiC loops had at least one anchor overlapped with active CREs, indicating their central roles in distal loop formation. Aggregate peak analysis (APA) (27) showed that loop strength fold enrichment for Tri-HiC was highest (20-fold) for CTCF loops and lowest for active enhancer loops (6.2-fold) (Fig. 3*D*). These numbers were substantially higher than typically obtained from in situ Hi-C (onefold to fourfold) (3), with the APA for the same loops of in situ Hi-C showing only positive, but weak, loop signal (1.1-fold) for CTCF loops and no loop signals for all other CRLs. At 200 bp resolution, we observed that the average loop size was typically about 1 × 1 kb (2), suggesting that subkilobase resolution is required for characterizing the loops without convoluting their signals with the background.

To evaluate the sensitivity of Tri-HiC CRL detection, we compared the virtual 4C profile of *MLLT3* from the method with Tri-4C (Fig. 3*E*). Out of 62 intra-TAD CRLs detected by Tri-4C, Tri-HiC identified 31 (50%) compared to 4 (6%) by in situ Hi-C (Fig. 3*F*). We examined 6 additional promoters located around multiple enhancers and found that the virtual 4C peaks generally well overlapped with DNase and H3K27Ac peaks, in contrast to the flat signals from in situ Hi-C (*SI Appendix*, Fig. S15*B*). Taken together, the improved resolution of Tri-HiC proved to substantially increase the detection of loops mediated by all types of active CREs genome-wide.

### Revelation of High Nonspecific Distal Interactivity of Regulatory Elements.

**Fig. 4.**
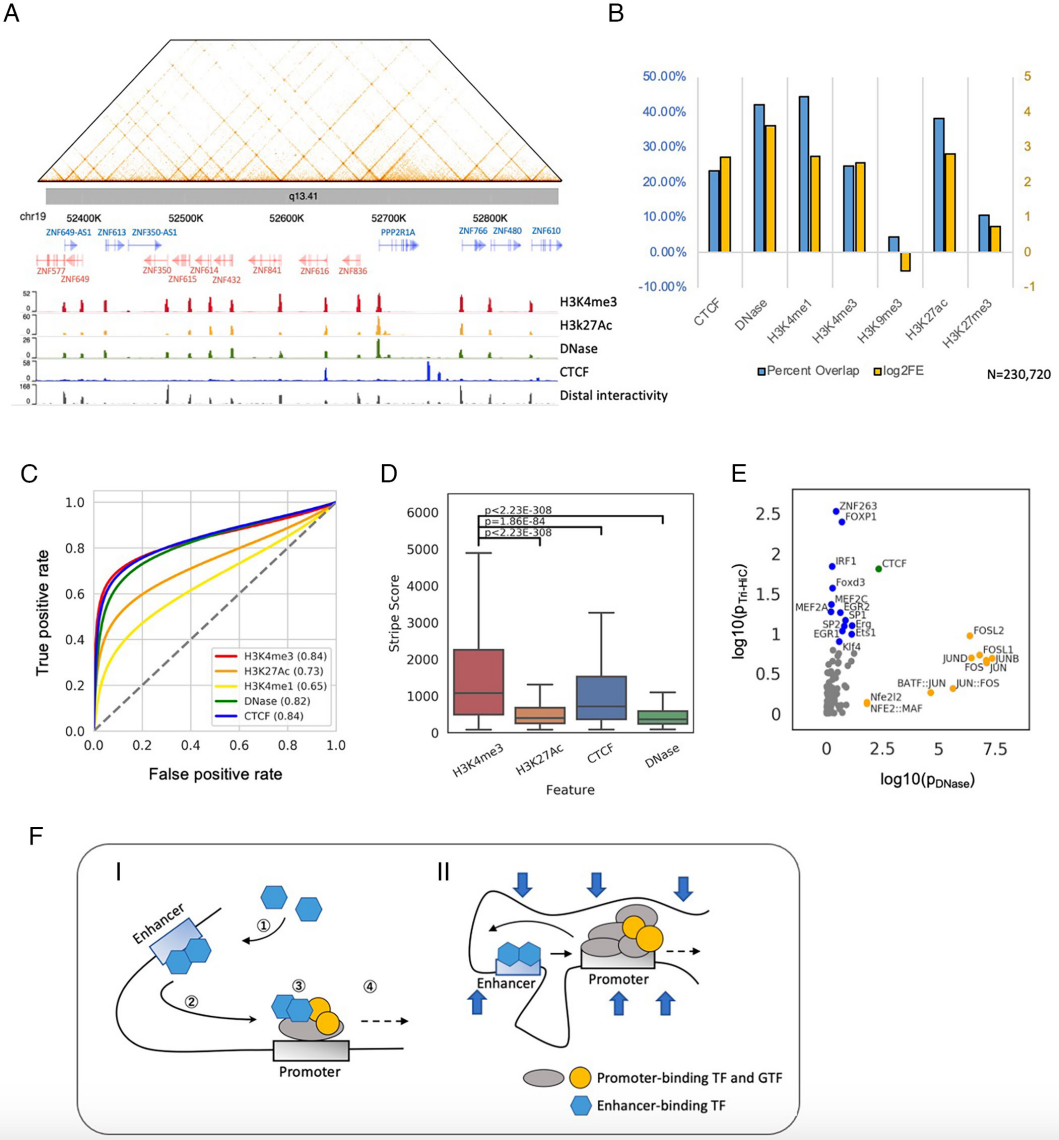
Tri-HiC identifies regulatory elements as distal interaction hotspots. (*A*) An example of gene promoters forming bidirectional nonspecific interaction stripes in chr19. Aligned with the landscapes of epigenetic marks, the distal interactivity track at the bottom indicates −log10 *P* value of distal interaction read count enrichment (*Materials and Methods*). (*B*) Percentage overlap and log2 fold enrichment (log2FE) of distal interaction hotspots identified by Tri-HiC with other 1D epigenetic marks. The interaction hotspots annotated into multiple categories are only included in the leftmost category. (*C*) ROC analysis using distal interactivity scores [−log(p)] for each 100 bp bin steps as predictors of CRE peaks. (*D*) Comparisons of distal interactivity scores for hotspots annotated with different epigenetic marks. Significance of differential interaction scores was calculated by using a two-sided *t* test. (*E*) Comparisons of motif enrichment scores (log10 *P* value) between IMR90 DNase peaks and distal interaction hotspots identified by Tri-HiC. (*F*) Hypothetical models interpreting the asymmetric insulation of promoter-enhancer loops. i) In the “transfer”/asymmetric loop extrusion model, ① enhancers recruit transcription factors and cofactors which ② mediates nonspecific interaction stripes before encountering the promoter. ③ Upon the contact, these factors are partially transferred from the enhancer to the promoter, ④ thereby weakening the enhancer interaction stripe downstream. ii) In the E:P retention model, enhancers are retained after meeting the preferred promoter. The large scaffold complex at the promoter thus blocks the enhancer from interactions further downstream, while the TF complex on the enhancer is not sufficiently large to prevent the promoter from interacting with the upstream target.

Tri-HiC marked loci that formed strong nonspecific interactions, visualized as stripes which extended up to hundreds-thousands kb (Fig. 4*A*). These stripes resulted in significant overrepresentation of distal interaction read counts, which we could identify using an algorithm similar to the loop caller for Tri-4C (*Materials and Methods*). With a stringent Bonferroni threshold (*P* < 3E-7), we identified 230,720 significant distal interaction hotspots at 100 bp resolution. Annotation of these hotspots indicated their large overlap with all active CRE epigenetic markers (Fig. 4*B*), with the highest being 12.2-fold enrichment with DNase, suggesting that high distal interaction activity is a general property of active CREs. Consistent with this interpretation, the distal interactivity score was strongly predictive of all active CRE markers, with the highest AUC scores found with H3K4me3 (0.84), CTCF (0.84), and DNase (0.82) (Fig. 4*C*). Quantitative comparisons of the interactivity scores among different CREs showed that promoter regions (H3K4me3) were the strongest distal-interacting elements, followed by CTCF binding sites, and both were higher than that observed active enhancers (marked by H3K27Ac) and other DNase-marked CREs (Fig. 4*D*). We further compared the motif enrichment between interaction hotspots and DNase peaks and found that DNase peaks were specifically more enriched with AP-1 class motifs, which have been reported to be depleted in promoters (29). In contrast, Tri-HiC interacting regions were more enriched with several CG-rich motifs, including SP1/2, EGR2, and KLF4 (Fig. 4*E*). These results together indicated that promoters were a highly enriched distal interactive element among all CREs.

Interaction hotspots were depleted in heterochromatin marked by H3K9me3, but instead enriched in H3K27me3-marked polycomb-repressed regions (Fig. 4*B*). Consistently, we observed interaction stripes from polycomb-repressed genes with strikingly long widths typically greater than 10 kb, suggesting that polycomb repression failed to prevent distal chromatin interactions (*SI Appendix*, Fig. S16). Notably, despite their extended widths, which allowed visualization at 2 to 20 kb resolution range in Tri-HiC, these super stripes were not detected by in situ Hi-C.

### Quantitative Analysis Dissects the Correlation between Loop and Stripe Formation.

Annotation of Tri-HiC loop anchors with distal interactivity indicated that the majority of loop anchors were also interaction hotspots (*SI Appendix*, Fig. S15*C*), which is consistent with our findings that CREs often loop with multiple partners within the stripe range, forming interaction networks such as the promoter network in the chr19 KRAB zinc finger protein cluster shown in Fig. 4*A*. Such observation raised the question whether the loops identified by Tri-HiC indicate a stable loop structure between CREs that has been widely referred in the enhancer-mediated transcriptional mechanisms (30) or merely a high incidence of colocalization between loci with high distal interactives. The two scenarios can be distinguished by quantitative analysis to determine whether the interaction frequencies observed at loops are higher than expected from the nonspecific interactions from the hotspots. By comparing the loop strengths to the product of the neighboring stripe strengths from the two loop anchors, we found that although they were significantly correlated (R = 0.67, *SI Appendix*, Fig. S17*A*), the loop strength was consistently higher for all types of CRLs, with the sole exception of promoter-promoter interactions (*SI Appendix*, Fig. S17*B*).

To further understand factors contributing to the CRLs, we deduced the loop strengths contributed from the stripe fold enrichments, and associated the residual loop strength with the motif component on the loop anchors. Hierarchical clustering of motifs showed that TFs in several families, including STAT, ETS, E-box, and AP-1, interacted stronger with the same family member and tended to have similar interaction preferences (*SI Appendix*, Fig. S17*D*). On the other hand, the CpG-associated motifs, such as ZBTB33, NRF1, and E2F, were associated with weaker strength regardless of the loop partner. Importantly, we found that the majority of motifs formed stronger interactions when paired with themselves (*SI Appendix*, Fig. S17*C*), which could implicate a general homotypic interaction of TF-bound regulatory regions. Although further experiments are required to clearly interpret these observations, Tri-HiC suggests that homotypic TF pairing is likely to play a significant impact on the loop strength.

### Insulator Properties Associated with Cis-Regulatory Elements.

The Tri-HiC interaction heatmap suggested non-CTCF-bound CREs segregate TADs into further microdomains (Fig. 2*A*). Consistently, aggregative analyses showed insulation of background interactions at active CREs similar to CTCF (*SI Appendix*, Fig. S18*A*). This finding was further confirmed by insulation score analysis (31), which indicated precise alignments between boundary signal peaks and interaction hotspots (*SI Appendix*, Fig. S18*B*). Quantification of insulation scores showed comparable values between CTCF and promoter regions (H3K4me3), both of which were significantly higher than active enhancers (H3K27Ac) and other CREs (DNase) (*SI Appendix*, Fig. S18*C*). These results suggested that while all CREs contributed to chromatin domain segregation, promoter regions exerted particularly strong insulation/retention effects that were analogous to CTCF sites typically found at TAD boundaries.

To assess the impact of microdomains on distal interactions between CREs, we analyzed the changes in interaction around CRLs by quantifying the intensities of the corners and stripes inside and outside the loops. We found that, similar to their effect on background, the promoters and CTCF-bound regions were insulative to their loop partners, reducing their stripe extension to the exterior of the loop (*SI Appendix*, Fig. S18*D*). By contrast, CREs such as active enhancers did not prevent the loop partner from interacting further downstream, although weak insulation of the background was observed. Thus, the differential insulation property of promoters vs. enhancers resulted in their asymmetric loop structure, where promoters could be construed “block” the enhancer from farther distal interactions but not vice versa (*SI Appendix*, Fig. S18*E*), consistent with ideas that fine-gauge E:P (enhancer:promoter) interactions are dynamically favored at the expense of other distal E:P pairing in the same contact domain (32).

## Discussion

We developed Tri-4C and Tri-HiC to further address the resolution limitations of current 3C methods. We demonstrated that the usage of a combination of multiple REs was essential and sufficient to capture interactions missed by the currently existing methods, thereby allowing comprehensive and quantitative identification of cis-regulatory loops at a one hundred basepair resolution. The approach of utilizing high-resolution analysis to obtain the true signal-to-background ratio of CRLs is distinct from enrichment-based approaches like immunoprecipitation such as HiChIP and PLAC-seq (9, 18), and avoids inevitable biases introduced by all enrichment strategies. The principle of multidigestion can be applied to these methods to further enhance their resolution and loop detectability. The multidigestion also improved the contact yield efficiency, which together with the Tn5 tagmentation strategy reduced the input DNA for Tri-HiC down to hundred nanogram range. Despite the known mild GC preference (33, 34), Tn5 has been widely used to uniformly fragment genomic DNA for low input high throughput sequencing. Potential tagmentation bias can later be addressed through normalization against the self-ligation contacts, which account for the majority of the Tri-HiC contacts (*SI Appendix*, Fig. S13*B*), to avoid false calling of significant distal interactions. These improvements together enabled Tri-HiC to be performed with as few as 0.1 million cells. Such a low input requirement should broaden the applicability of the method to samples with limited availability, extending its potential clinical utility.

With improved resolution, we found that CREs played central roles in organizing distal chromatin interactions, uncovering finer chromatin structural events within the TAD structures implicated by Hi-C assays. Tri-HiC indicated that active CREs formed both nonspecific stripes with neighboring regions and loops with other CREs, which corresponded with the loops identified by Tri-4C. These analyses suggested that direct loop interactions with promoters can interpret the transcriptional alterations caused by mutations of distal CREs lacking CTCF and cohesin binding. Our result also suggested that high distal interactivity can be used as a parameter to map CREs, providing examples by using Tri-4C to identify functional regulatory elements. The AS looping detected by Tri-4C, as well as the quantitative correlation between loop strength and ATAC-seq peak strength, indicated a correlation between distal interactivity and activities of CREs, which is consistent with recent studies showing linkage between enhancer gain and loop formation during cell differentiation (8, 35). However, the revelations of super-stripes at polycomb-repressed genes by Tri-HiC suggest that distal interactivity does not fully depend on the activation properties of the CREs.

The association of CREs with high insulation activities suggests their involvement in segregating TADs into smaller microdomains. At TAD level, the domain formation has been well explained by the loop extrusion model in which the convergent CTCF pairs confined cohesins that mediated high background interaction within the TAD (36). However, the absence of measurable CTCF and cohesins at promoters and enhancers suggests distinct factors or mechanisms are maintaining the microdomains at these elements. Because Tri-HiC data suggests that both loop extrusion and homotypic E:E and E:P interactions (*SI Appendix*, Fig. S17*D*) are aspects of CRLs, it can be inferred that formation of CRLs should involve both the cohesive factor-mediated chromatin junction and interactions between motif-binding TFs. Tri-4C and Tri-HiC provide valuable tools for future studies to dissect the interplays between regulatory loops and TF interactions between regulatory elements.

In contrast to the symmetric insulation on both ends of TAD boundaries, we found that enhancers were insulated in an asymmetric fashion by their looped promoters. To interpret such observations, we proposed two hypothetical models (Fig. 4*F*): in a one-sided loop extrusion model, the P-E loop contact results in the enhancers transferring some of the interaction-mediating/activating machinery to the promoter upon looping, which is stabilized and reduces their own distal interactivity; or an E:P retention model, enhancers are preferentially retained, likely in a cohesin-dependent fashion, to a preferred promoter in the contact domain, thereby retaining their interactions. Both models suggest that promoters can decrease the ability of an enhancer to interact with the less-favored potential promoter targets, which is supported by recent findings that the long noncoding RNA PVT1 is an insulator for its upstream gene *MY*C (32). We found the promoter of *PVT1* “inhibited” the extension of the interaction stripes for all its intronic enhancers, including four with validated function, to *MYC*, consistent with the concept of retention of enhancer interactions with a preferred cognate promoter (*SI Appendix*, Fig. S18*F*). Interestingly, we identified multiple oncogene regions resembling the *MYC* locus where lncRNAs resided between the oncogene and its looped enhancer clusters (*SI Appendix*, Fig. S19). However, the insulation effect was only found on active and stripe-forming lncRNA promoters such as *LINC01798* in the *MEIS1* locus. Thus, the preferred promoter does not always overlap with enhancer proximity.

## Materials and Methods

### Cell Culture.

IMR90 cells (ATCC CCL-186) were maintained in Eagle’s Minimum Essential Medium (EMEM) (Corning 10-009-CV) with 10% fetal bovine serum (GEMINI 100-500). To induce *IFNB1* expression, cells were treated with 20 µM 2′3′-cyclic GMP-AMP (Invivogen tlrl-nacga23), 100 ng/mL IFNγ, and 10 ng/mL TNFα for 24 h (20, 37). Cells were collected at full confluence for all downstream analyses.

Human embryonic stem cells H7 (WiCell WA07) were maintained in a feeder-free E8 system (ThermoFisher A1517001). Differentiation of the ES cells to VSMCs was conducted as previously described (38). Briefly, cells were plated on vitronectin (ThermoFisher A14700) coated surface at 5 to 10% density. On day 2, the medium was switched to N2B27 [50% DMEM-F12 + 50% Neurobasal medium + 1× N2 supplement (ThermoFisher 17502048) + 1× B27 supplement (ThermoFisher 17504044)] supplied with 10 µM CHIR-99021 and 25 ng/mL BMP4 to induce mesoderm differentiation. From day 5, cells were incubated in N2B27 medium supplied with 10 ng/mL PDGF-BB (Peprotech 100-14B) and 2 ng/mL Activin A (Peprotech 120-14P) to induce VSMC differentiation. Five days later, Activin A was retrieved from the medium, and the cells were expanded for two population doublings and collected for analysis.

### RNA Isolation and qRT-PCR.

RNA was isolated from cells using the PureLink RNA mini kit (Thermo 12183020) according to the manufacturer’s instructions. DNase treatment was performed using the DNA-free DNase Treatment & Removal Kit (Thermo AM1906). To synthesize cDNA, reverse transcription was performed using SuperScript IV Reverse Transcriptase (Thermo 18091050) with oligo-dT primers following the manufacturer’s instructions. Quantitative RT-PCR was performed using the Applied Biosystems StepOne Plus platform. For *IFNB1*, a predesigned Taqman probe (Hs01077958_s1) was used with TaqMan Universal PCR Master Mix (Thermo 4304437). For *MLLT3*, a custom primer pair (Fw: TTTGTGGAGAAAGTCGTCTTCC; Rev: GAGGTGATTCACTGGTGGATG) was used with PowerUp SYBR Green Master Mix (Thermo A25741). Expression was quantified using the delta CT method, normalized to *HPRT1* (Thermo 4326321E).

### Cas9-Mediated Gene Editing.

Guide RNAs for the Cas9 endonuclease were selected using the CRISPR design tools from Zhang lab (http://crispr.mit.edu). To generate enhancer deletions in IMR90 cells, the Integrated DNA Technologies (IDT) ribonucleoprotein (RNP) system was applied following the protocol provided by the manufacturer. Briefly, synthesized crRNAs were annealed with tracrRNA and incubated with Cas9 V3 (IDT 1081058) at equimolar concentrations. To perform NHEJ-mediated deletion, we transfected 1 × 10^5^ IMR90 cells with 22 pmol of Cas9 RNP using the Neon electroporation system with resuspension buffer R (ThermoFisher) at 1,100V, 30 ms, 1 pulse. After 72 h, cells were collected for genomic DNA and RNA extraction. To measure deletion efficiency, target sites were amplified using the validation primers that flank the deletion region (as indicated in Dataset S3) and were examined using electrophoresis.

### ATAC-seq.

ATAC-seq was performed as previously described, with minor modifications (21). Briefly, 50,000 IMR90 cells were collected and resuspended in 50 µL cold lysis buffer [10 mM Tris-HCl pH 8.0, 10 mM NaCl, and 0.2% Igepal CA630 (Sigma)]. After incubation on ice for 5 min, cells were centrifuged at 800 x g for 5 min at 4 °C. The cell pellet was resuspended with 50 µL transposition mix containing 25 µL 2× TD buffer (Illumina FC-121-1030), 3.5 µL Tn5 transposase (Illumina FC-121-1030), and 21.5 µL water. The mixture was incubated at 37 °C for 30 min with gentle rotation, and transposed genomic DNA was recovered using DNA Clean & Concentrator-5 (Zymo Research D4013). The library was amplified using NEBNext high fidelity PCR master mix (M0541) containing 1.25 µM customized Nextera universal (Ad1_noMX) and indexed primer. The cycling condition was set to 72 °C for 5 min, followed by 10 cycles of 98 °C for 30 s, 63 °C for 10 s, 72 °C for 1 min, and a final extension at 72 °C for 5 min. The amplified library was purified using SPRI beads (Beckman B23318). A double size selection was performed with 0.5×/1.8× bead volume to remove amplicons >1,000 bp or <100 bp. Libraries were subjected to 150 bp pair end sequencing on the Illumina HiSeq platform with an expected read depth of 70 million. The FASTQ data were aligned and analyzed using the pipeline from the Kundaje Lab (GitHub https://github.com/kundajelab/atac_dnase_pipelines).

For ATAC-dPCR for ECAD9, 20 ng of the final library was loaded into the Quantstudio 3D digital PCR system (version 2, ThermoFisher) and amplified using Taqman array (rs4977575, ThermoFisher C__27869497_10). AS signal quantification was performed using the online cloud application provided by the manufacturer.

#### Tri-4C and Single RE UMI-4C Library Construction.

To generate the preamplification library, Tri-4C adapted the in situ Hi-C and UMI-4C protocols (7, 10). 10^7^ cells were fixed with 1% (v/v) formaldehyde (Thermo Fisher 28906) in PBS for 10 min at room temperature. Fixation was quenched by adding 2.5 M glycine, dropwise, to a 0.2 M final concentration and incubating for 5 min at RT. Cells were washed with cold PBS twice and pelleted (300 x g, 4 min, 4 °C) in 2 mL Eppendorf LoBind tubes. Pellets could be immediately used for downstream procedures or snap-frozen with liquid nitrogen and stored at −80 °C.

To prepare crude nuclei, the cell pellet was resuspended in a premixture of 250 µL cold lysis buffer [10 mM Tris-HCl pH 8.0, 10 mM NaCl, and 0.2% Igepal CA630 (Sigma)] and 50 µL protease inhibitors (Sigma P8340). After mixing thoroughly, the suspension was incubated on ice for 15 min and centrifuged (1,000 x g, 5 min, 4 °C). The pellet was washed once with 500 µL of cold lysis buffer and carefully resuspended in 50 µL of 0.5% sodium dodecyl sulfate (SDS). The suspension was then incubated in a 62 °C heating block for 7 min, followed by mixing with 145 µL water and 25 µL 10% Triton X-100 (Sigma), and incubated at 37 °C for 15 min for quenching. To carry out triple digestion, the suspension was mixed with 50 µL of buffer G (ThermoFisher), 120 U MboI (DpnII) (Thermo Fisher ER0811, 10 U/µL), 120 U Csp6I (CviQI) (Thermo Fisher ER0211, 10 U/µL), 100 U Hin1II (NlaIII) (Thermo Fisher ER1831, 5 U/µL), and x µL of water, where x is determined to bring the total volume to 500 µL, empirically within a range of 100 to 150 µL, depending on the pellet size.

The genomic triple digestion can be alternatively performed by using a combination of MboI (Thermo Fisher), Csp6I (Thermo Fisher), and CviAII (New England Biolabs) to generate consistent 5′ TA overhangs for other 3C-derived protocols requiring biotin-dA filling. In this case, after Triton quenching, the nuclei suspension was mixed with 50 µL of 10× CutSmart Buffer (NEB) and 100 U CviAII (NEB) and diluted to 500 µL. The mixture was incubated at 25 °C with rotation for 2 h and then 37 °C for 2 h or overnight after adding 120 U MboI and 120 U Csp6I.

For Single RE UMI-4C (11) experiments, the suspension was mixed with 50 µL of buffer R, buffer B, and buffer G, respectively, for digestion using 100 U MboI, 100 U Csp6I, or 100 U Hin1II in 500 µL final volume. All digestions were conducted at 37 °C overnight with rotation.

On the second day, the REs were inactivated by incubating at 65 °C for 20 min. After cooling to room temperature, end blunting was performed by adding 3 µL of 10 mM dNTP, 8 µL of DNA polymerase I Klenow (NEB M0210, 5U/µL), and 4 µL of T4 DNA polymerase (NEB M0203, 3 U/µL) and incubating at 37 °C for 1 h with rotation. For blunt end ligation, the suspension was then mixed with 460 µL water, 120 µL T4 ligase buffer (NEB B0202), 100 µL 10% Triton X-100, 6 µL 20 mg/mL BSA (NEB B9000S), and 5 µL of 400 U/µL T4 ligase (NEB M0202S/L) and incubated for 4 h at room temperature with rotation. The processed nuclei were pelleted (1,000 x g, 5 min, 4 °C) and resuspended in 500 µL 1× T4 ligation buffer supplemented with 50 µL 20 mg/mL proteinase K (Thermo AM2546) and 50 µL 10% SDS and incubated at 55 °C for 30 min. For de-crosslinking, 60 µL 5 M sodium chloride was added, and the mixture was incubated at 68 °C for 2 h. Note that this step can also be prolonged to overnight. Phenol–chloroform extraction was performed to recover DNA as follows: The suspension was washed once with an equal volume of Phenol:Chloroform:Isoamyl alcohol (25:24:1), and once with an equal volume of Chloroform:Isoamyl alcohol (24:1). After phase separation, the aqueous phase was transferred to a new 2 mL LoBind Eppendorf tube, mixed with 60 µL 3 M sodium acetate, 1 µL GlycoBlue coprecipitant (Thermo AM9515) and 1.5 mL pure ethanol, and incubated at −80 °C for 15 min. The mixture was centrifuged at max speed at 4 °C for 15 min. The supernatant was carefully removed, and precipitated DNA was washed twice with 1 mL cold 70% ethanol. The DNA pellet was air dried for 15 min and resuspended in 130 µL 10 mM Tris-HCl (pH 8.0) for 1 h at room temperature or overnight at 4 °C.

The rearranged genomic DNA was sonicated to 300 to 400 bp fragments using a Covaris S2 ultrasonicator. The parameter guidelines from the manufacturer were used, with settings of Intensity (4), Duty cycle (10%), cycles per burst (200), and time (80 s) as starting points. In general, multiple rounds (typically 2) with the above parameters were run to obtain the desired fragment size peak of 300 to 400 bp, which was confirmed by Bioanalyzer (Applied Biosystems). The fragmented DNA was double size-selected using 0.40×/1.0× of SPRI beads (Beckman) to remove fragments below 100 bp and above 1,000 bp, and eluted in a final volume of 70 µL of 10 mM Tris-HCl. To repair the sonicated fragment ends, the eluent was mixed with 10 µL 10× T4 ligation buffer (NEB), 10 µL 100 mM ATP, 5 µL 10 mM dNTP mix, 4 µL T4 DNA polymerase (NEB), 1 µL DNA polymerase Klenow (NEB), and 5 µL T4 PNK (NEB M0201, 5 U/µL) and incubated for 30 min at room temperature. The repaired DNA was purified by using 1.0× SPRI beads and eluted in a master mix of 94.5 µL 1× NEB buffer 2 and 0.5 µL 100 mM deoxyadenosine triphosphate. After removing the beads, 5 µL of Klenow exo- (NEB M0212S/L, 5 U/µL) was added, and the mixture was incubated at 37 °C for 30 min for dA tailing. The processed DNA was purified by using 1.0× SPRI beads and eluted in 20 µL 10 mM Tris-HCl.

We designed a custom Y-shaped adaptor to generate Illumina next-generation sequencing libraries:

Forward: G​ATC​TAC​ACT​CTT​TCC​CTA​CAC​GAC​GCT​CTT​CCGATC*T
Reverse: /5Phos/GATCGGAAGAGCCATACAGC

The oligos were synthesized using the IDT Ultramer service (Integrated DNA Technologies). The forward and reverse single-strand oligos were annealed (95 °C for 5 min, down to 25 °C at 0.1 °C/s temperature gradient) and prepared at 30 µM stock concentrations. Five µL of adapter was added to the A-tailed libraries and mixed with 25 µL blunt/TA ligase master mix (NEB M0367). The mixture was incubated at room temperature for 15 min and purified with 1.0× SPRI beads. After eluting in 50 µL Tris-HCl, the libraries were purified a second time with 1.0× SPRI beads to completely remove the residual adaptors. The final preamplification libraries were eluted in 100 µL Tris-HCl and examined by Bioanalyzer (Applied Biosystems) to ensure correct size distributions and absence of unligated adaptors. The size distributions of mature libraries after incorporating adaptors were centered around 500 bp.

To generate the final Tri-4C and single RE UMI-4C libraries, we designed a pair of outer and inner primers, based on the RE, for each viewpoint to increase amplification specificity (Dataset S1). For amplification with outer primers, eight 100 µL reactions, each containing 400 µg preamplification library, 2 µM universal primer (AATGATACGGCGACCACCGAGATCTACACTCTTTCCCTACACGACGCTC), 0.5 µM viewpoint-specific outer primer for each multiplexed viewpoint, and 1× SuperFi PCR master mix (Thermo 12358010) with 20% GC enhancer, were amplified with the following conditions: 98 °C for 30 s, 14 cycles of 98 °C for 10 s, 62 °C for 10 s, and 72 °C for 60 s, and final extension at 72 °C for 5 min. All primers were synthesized using the IDT Ultramer service (Integrated DNA Technologies). The products were pooled and purified with 1.0× SPRI beads, and amplified with the inner primer pair (Illumina P5 + bait-specific P7 index-attached reverse primer) for 14 cycles using the same conditions. After purification with 1.0× SPRI beads, the products were quantified using the Qubit DNA assay kit (Thermo Q32851), examined by Bioanalyzer (Applied Biosystems), and diluted to 10 nM to be sequenced on Illumina platforms.

We aimed for a read depth of 5 million reads for each viewpoint. For a typical library containing 100,000 unique fragments, this results in 50× coverage. The high coverage is desired as Tri-4C generates more reads than single RE UMI-4C with the same DNA input. In practice, the actual yield varies in multiplexed libraries, possibly due to primer efficiency and off-target amplification. A minimum depth of 1 million reads was required for all our experiments. Sequencing was performed on Illumina platforms (MiSeq/HiSeq) in paired read mode with read lengths of 75 to 150 bp.

### Tri-HiC Library Construction.

The initial cross-linking and cell pellet preparation procedures for Tri-HiC were the same as the Tri-4C protocol, with the exception that each pellet included 80 to 240 thousand cells. The pelleted cells were treated with 250 µL cold lysis buffer with 50 µL protease inhibitors and washed once with 500 µL of cold lysis buffer. The crude nuclei were permeabilized by resuspending in 200 µL of 0.5% Triton X-100 in lysis buffer and incubating at room temperature for 15 min. To carry out triple digestion, the nuclei were then pelleted by centrifugation and resuspended in 250 µL of master mix containing 0.1% SDS, 1× CutSmart Buffer (NEB), 10 µL MboI (NEB R0147S), 5 µL CviQI (NEB R0639S), and 5 µL CviAII (NEB R0640S). The digestion was performed at room temperature for 2 h and then 37 °C for 2 h. The REs were then inactivated by incubating at 65 °C for 20 min. After cooling to room temperature, end blunting was performed by adding a 50-µL master mix containing 1.5 µL of 10 mM deoxycytidine triphosphate, 1.5 µL of 10 mM deoxyguanosine triphosphate, 1.5 µL of 10 mM deoxythymidine triphosphate, 37.5 µL of 0.4 mM biotin-deoxyadenosine triphosphate (ThermoFisher), and 4 µL of DNA polymerase I Klenow (NEB) and incubating at 37 °C for 1 h with rotation. For blunt end ligation, the suspension was then mixed with 200 µL of master mix containing 50 µL 10× T4 ligase buffer (NEB), 2.5 µL 20 mg/mL BSA, and 5 µL of 400 U/µL T4 ligase (NEB). The ligation was performed with rotation at room temperature for 2 h, then overnight at 4 °C, and additional 2 h at room temperature. The processed nuclei were pelleted and resuspended in 500 µl 1× T4 ligation buffer supplemented with 50 µL 20 mg/mL proteinase K and 50 µL 10% SDS, and incubated at 55 °C for 30 min. For de-crosslinking, 60 µL 5 M sodium chloride was added, and the mixture was incubated at 68 °C for 2 h or overnight. The DNA content was then purified by phenol–chloroform extraction, as described in the Tri-4C protocol, and resuspended in 15 to 50 µL 10 mM Tris-HCl, scaling with the input.

To construct the Tri-HiC Illumina library, we performed the following reactions defined in units. Each unit started with 300 ng purified proximity-ligated DNA quantified by Qubit (ThermoFisher), which should be obtained from <100 k starting cells. For tagmentation, one unit of input was diluted to 30 µL and mixed with 50 µL of 2× TD buffer and 20 µL of tagment DNA enzyme (TDE) (Illumina 20034197, Lot 20436911), and incubated at 55 °C for 10 min (Note: the amount of TDE depends on the storage condition of the enzyme. We recommend optimizing the exact amount to obtain fragments peaked at 250 to 400 bp range). The tagmented DNA was purified with DNA Clean & Concentrator-5 (Zymo Research) and eluted in 30 µl elution buffer. The eluent was mixed with 50 µl 2x NEBNext high-fidelity PCR master mix, 1.25 µM ATAC-seq primers, and diluted to 100 µL final volume to be amplified by a 2-cycle PCR with the cycling condition of 72 °C for 5 min, 2 cycles of 98 °C for 10 s, 63 °C for 10 s, 72 °C for 1 min, and final extension at 72 °C for 5 min. Meanwhile, 15 µL of 10 mg/mL Streptavidin C1 Dynabeads (ThermoFisher) was prepared by washing 3 times with 200 µL of 2× binding and washing (B&W) buffer (10 mM Tris-HCl pH 7.5, 1 mM EDTA, and 2 M NaCl). The beads were resuspended with 100 µL 2× B&W buffer and were directly added to the PCR product, mixed with pipetting, and incubated for 15 min at room temperature with rotation. Beads were then separated on a magnet and washed 3 times with 500 µL 1× B&W buffer (5 min incubation each), 1 time with 10 mM Tris-HCl, and finally resuspended in 100 µL PCR solution containing 50 µL 2× NEBNext high-fidelity PCR master mix and 0.5 µM ATAC-seq primers. A following PCR reaction was performed with the cycling condition of 98 °C for 30 s, 8 cycles of 98 °C for 10 s, 63 °C for 10 s, 72 °C for 1 min, and final extension at 72 °C for 5 min (Note: we recommend to remove beads from the reaction at the end of the 4th cycle). The amplified library was size-selected and purified by 0.55 to 1× SPRI beads before QC and sequencing.

For this study, we constructed 5 Tri Hi-C libraries (biological repeats) using IMR-90 cells, with one starting with 80 thousand, one with 160 thousand, and three 240 with thousand cells. Final libraries were pooled and sequenced in one single flow cell (expected 10 billion reads output) of the Illumina Nova-Seq S4 platform using the 2 × 100 bp pair-end sequencing.

### Data Analysis.

Detailed analysis procedures for Tri-4C and Tri-HiC are described in supporting information.

### Public Data Usage.

The following public datasets for IMR-90 were used for annotations and comparative analysis of Tri-4C and Tri-HiC: DNase (GSE18927), H3K4me1 (GSE16256), H3K4me3 (GSE16256), H3K9me3 (GSE16256), H3K27Ac (GSE16256), H3K27me3 (GSE16256), CTCF (GSE31477), Rad21 (GSE31477), SMC3 (GSE91403), in situ Hi-C (GSE63525), and ENCODE Transcription Factor ChIP-seq Clusters (wgEncodeRegTfbsClusteredV3 track).

## Supplementary Material

Appendix 01 (PDF)

Dataset S01 (XLSX)

Dataset S02 (XLSX)

Dataset S03 (XLSX)

Dataset S04 (XLSX)

## Data Availability

Raw and processed data are available at NCBI Gene Expression Omnibus (GEO), with accession numbers GSE119189 (39) and GSE161014 (40).
