# Supplementary material for: Ultrafine mapping of chromosome conformation at hundred basepair resolution reveals regulatory genome architecture
Source: Proc Natl Acad Sci U S A. 2023 Nov 3;120(45):e2313285120. doi: 10.1073/pnas.2313285120 (PMC10636305; doi:10.1073/pnas.2313285120)
Supplement: Supplementary file 1 — Appendix 01 (PDF) [file pnas.2313285120.sapp.pdf]

Tri-4C Data Analysis

Generation of contact probability map

Due to the utilization of the multiple restriction enzymes, most existing 4C pipelines are not applicable to Tri-4C. However, alignment and processing is straightforward, as described below. After demultiplexing, the reverse end (R2, viewpoint end) of the FASTQ file was used to filter reads that were correctly ligated with the viewpoint by matching the sequence head with the inner primer sequence and the padding sequence. We used FASTX Barcode Splitter (http://hannonlab.cshl.edu/fastx_toolkit/index.html) for this step, with an allowance of 1 mismatch. The tool also trimmed the viewpoint sequence during the process. For allele-specific analysis, the reads were split by matching the allele with the tag SNP on the padding sequence using an awk command. The undigested/unligated ratio was calculated at this step by measuring the fraction of trimmed reads, starting with the immediate downstream sequence from the viewpoint. After trimming the viewpoint portion, the remaining reverse end was mapped together with the forward end (R1, sonication end) by using BWA mem with default paired end alignment settings against the hg19 genome. The aligned reads were deduplicated according to the mapped position of the sonication end (5' for the reads on the + strand and 3' for the - strand) by using a simple AWK command. We considered reads with sonication ends separated by 1 bp as duplicates based on the observation that the Illumina platform occasionally bypassed the first nucleotide of the read. Reads with low quality (MAPQ = 0) were removed from analysis. The complexity of the library (number of unique reads) and intrachromosomal ratio were measured at this stage. Reads from 1 kb upstream to 2 kb downstream of the viewpoint were removed as these regions were consistently highly interactive and subjected to over deduplication due to saturation of unique sonication ends. Interchromosomal interactions were also excluded from most downstream analysis since no loop-like interaction hotspots outside the same chromosome were observed. For standard analysis, the processed reads were binned in 500 bp, with a sliding step of 100 bp for both visualization and other downstream analyses, with the exception of reproducibility tests (**Fig 1c, Fig S3c, d**), where reads were binned with the indicated bin size and equal step size. For comparison of the analysis at the conventional lower resolution, reads were binned in 3000 bp, sliding at 100 bp. Of note, we used the entire aligned sequence for this step, instead of assigning each read to its corresponding restriction site, for two reasons. First, we observed a clear directional bias on the target restriction sites, which indicated that the point of proximity was not at the RE site but in its up- or down-stream vicinity. Thus, a short overhang during alignment strengthened the signal on the side of the vicinity. Second, the Tri-4C method was designed for promoter/enhancer viewpoints which often contained high/low GC contents. The difficulty of designing primers for these regions sometimes results in a long padding sequence and an unmappable short residual sequence on the reverse end after trimming, yielding reads that cannot be matched to the corresponding RE sites. The piled raw read count bedGraphs were used to perform peak calling. To ensure fair comparison, single RE UMI-4C libraries were analyzed in parallel using the same pipeline.

Loop Peak Calling

We used a local fold enrichment-based strategy to identify significant interaction loop peaks for the Tri-4C and single RE UMI-4C data. Thus, the expected number of reads (background) for a given bin with read count M was estimated by taking the average of neighboring bins. We used the smallest mean value of 5 kb, 10 kb, 20 kb, and 50 kb intervals centered at the bin location, to represent the background N. Then, significance p values were calculated by $p = Pr\{X \geq M\}$ given $X \sim Poisson(N)$. Of note, this step can be achieved by feeding the MACS2 bdgcmp function with the background and signal tracks using –m ppois mode[18].

To identify significant and reproducible peak regions, bins were scored with the –log10(-log10(p)) value, and those with a score > 0 (p < 0.1) in all replicates were collected and analyzed by IDR package[19] (Github https://github.com/nboley/idr) using the following settings

--initial-mu 1.5 --initial sigma 0.3 --initial-rho 0.8

Bins with IDR < 0.05 (score ≥ 540) were considered significant and merged. We defined a minimum length of 300 bp for calling significant distal loop peaks.

The UMI-4C[11] and 1D adaptation of *in situ* Hi-C[3] loop calling algorithms were used for comparison. For both algorithms, distance-dependent decay of interaction frequency was calculated at genome-wide level using IMR90 *in situ* Hi-C data at 5 kb resolution. The decay function was further smoothed to 500 bp bins (W) in 100 bp step size resolution by using linear interpolation to obtain the $F(d)$ (UMI-4C) or E^* (Hi-C) suitable for high resolution analysis in Tri-4C. For the UMI-4C algorithm, the background of each Tri-4C profile was obtained by re-allocating the total intra-TAD Tri-4C read counts (N) to each bin according to $F(d)$. The enrichment p value of a bin with expected read count of E and actual count of E_1 was calculated by fitting to binomial distribution: $Pr(B(N, E/N) > E_1)$. For the 1D *in situ* Hi-C algorithm, the adjusted expected read count for each bin $E^{d^*}{}_i$ can be calculated by the filter formula

$$E_i^{d^*} = \frac{\sum_{a=i-w}^{i+w} M_a^* - \sum_{a=i-p}^{i+p} M_a^*}{\sum_{a=i-w}^{i+w} E_a^* - \sum_{a=i-p}^{i+p} E_a^*} \times E_i^*$$

Where p and w were set at 2 and 100 to be corresponding with 500 bp peak and 20 kb background size. This expected count was compared with actual read count M_i using the Poisson statistics. The p values obtained from UMI-4C and Hi-C algorithms were directly compared with Tri-4C raw p values by the CRE ROC analysis.

To investigate for potential artifacts during Tri-4C loop calling due to mapping bias, the GC content and restriction site density under triple enzyme digestion for the locus analyzed by Tri-4C were obtained by directly analyzing the hg19 genome sequence of the region. Mappability of the region was obtained from the ENCODE mappability track available on the USCS genome browser. The average GC content, restriction site density, and mappability for the 10 kb neighboring regions for all loop sites called by Tri-4C were calculated at 100 bp resolution. To generate the background for comparison, a set of 10,000 equal size genomic intervals were randomly selected in the locus. The mean for each set was calculated after removing intervals whose center fell within Tri-4C loops or repeat regions.

Data Reproducibility

Each Tri-4C and single RE UMI-4C was performed in two technical replicates. Reproducibility of intrachromosomal interaction was measured by Pearson's correlation r using the Python numpy.corr function after binning the contacts in the size described in **Fig S1c**.

Analysis of Interaction Frequency and Loop Strength

For frequency-based analysis, the read count for each bin was converted to interaction frequency by normalizing against (1) total intrachromosomal interactions, which we referred to as normalized interaction or (2) total intrachromosomal interactions of a reference viewpoint in a multiplexed run. We refer to this as relative interaction frequency. The purpose of the latter method was to control for the significant change in total read count generated between different experimental conditions,

as in the *IFNB1* viewpoint at the baseline control compared to the *IFNB1* induced condition (**Fig S10c,e**). Thus, the reference point was selected based on the standard of exhibiting the least variation in unit read count yield among different conditions, and in our experiment the Boundary viewpoint was chosen for that reason. Subtraction analysis was performed by directly calculating the interaction frequency difference between two tracks in comparison after normalization. A multiplication factor of 10,000 was applied to the normalized interaction frequency to simplify the display when presenting the interaction map on UCSC/WashU track.

For loop-based analysis, loop strength, i.e. log fold enrichment (logFE) was calculated by $logFE = log10(M/N)$, where M and N are the actual and expected read count for each sliding window as indicated in the Peak Calling section. This step can be performed with MACS2 using –m logFE. A 1.0 pseudo count was given to calculate logFE to resolve zero division as well as attenuating noise level at regions with sparse mapped counts. Differential loop strength was calculated by measuring the logFE difference between two tracks. For presentation in **Fig S10e**, the logFE was weighed by frequency at basal condition.

Allele-specific Loop Calling

We used a likelihood ratio test, based on the loop strength, to determine whether an interaction loci displayed allelic bias. Specifically, for each 100 bp sliding window we obtained four vectors: M_{ref}, M_{alt}, N_{ref}, and N_{alt}, where *ref* and *alt* denoted the allele genotype, and M and N denoted the actual and expected read count, as indicated above. Each element in the vector represented one replicate. Firstly, the elements in N_{ref} and N_{alt} were normalized to their respective mean, and the scalars were used to normalize their corresponding M. Then, we had H_1: $\hat{M}_{ref} \sim Pois(\hat{N}_{ref})$; $\hat{M}_{alt} \sim Pois(\hat{N}_{alt})$, and H_0: $\hat{M}_{ref} \sim Pois(N_0)$; $\hat{M}_{alt} \sim Pois(N_0)$, where N_0 denotes the mean of $\hat{N}_{ref}$ and $\hat{N}_{alt}$. The likelihood ratio L was converted to p value by applying the Wilks' theorem, namely $-2ln(L) \sim \chi^2(1)$, subjected to subsequent Bonferroni correction where n equal to total number of assayed intervals in the locus.

Analysis of Cis-regulatory Element Interaction Network

To annotate CREs and active enhancers, respectively, DNase and H3K27Ac peak position and intensity for IMR90 cells were obtained from the Roadmap Project web portal (https://egg2.wustl.edu/roadmap/web_portal/). For the comparison of loop strength and interaction frequency between viewpoints with DNase peak intensity, linear regression models were built in Python using the scipy.stats.linregress function.

Receiver Operating Characteristic (ROC) Analysis

CRE positions, defined by Roadmap DNase peaks, were converted to 100 bp 1/0 tracks, with 1 indicating the presence of peaks inside the bin. The ROC curves were built by using loop scores (-log(p)) obtained from Tri-4C and UMI-4C as predictors for the peak positions in the converted DNase track, using Python sklearn.metrics: roc_curve and auc function with default settings.

Motif Analysis

The DNA sequences for all Tri-4C peak regions in both baseline control and *IFNB1*-induced conditions were extracted. Motif prediction was performed using TFBSTools (R platform) with accession to the JASPAR2018 database[39,40]. A minimum score of 90% was set to discover matched motifs. The Lasso CV model (CV=10, iter=10,000) was applied to all motifs to identify factors correlated with ΔlogFE of Tri-4C peaks during induction. Significance of correlation was determined by F statistic and subject to Bonferroni correction.

Hi-C and Topologically-Associated Domain (TAD) Definition

The *IFNB1* TAD (chr9:19480000-2120000) was defined by the *in situ* Hi-C data of IMR-90[7]. Hi-C Browser (http://promoter.bx.psu.edu/hi-c/index.html) was used to visualize the Hi-C interactions in the TAD.

Code Availability

The Shell pipeline used to align Tri-4C data and interaction loop analysis is available on Github (https://github.com/kimagure/Tri-4C).

Tri-HiC Data Analysis

Contact Map Alignment

The raw Fastq for Tri-HiC was aligned to hg19 genome and processed to obtain the interaction contact matrix in .mcool format by applying the Distiller pipeline[28] with default configurations at resolutions of 100, 200, 500, 1000, 2000, 5000, 10000, 20000, 50000, 100000, 250000, 500000, and 1000000 bp. The pairsam intermediate output from Distiller was processed by Juicebox Pre[27] to generate contact maps in .hic format with the same resolution setup. We kept both the alignments with filtering of MAPQ > 0 and > 30. For all analyses downstream we chose MAPQ > 30 by default, although in regions reported from this study, we did not observe distinguishable differences between the two parameters. For this study, we used HiGlass[41] to visualize the contact matrices in .mcool format.

Reproducibility Test

Reproducibility of Tri-HiC at 500 bp to 20 kb resolution range (**Fig S13a**) was evaluated by the Pearson correlation coefficient between replicates calculated by the HicCorrelate function in the HiCExploer[42] package. Due to the sparsity of distal contacts at extreme long range which results in mostly empty bins for the high resolutions in our analysis, we restrained the test to 8 Mb intrachromosomal range, which is in line with the distance limit for loop calling (see sections below).

Decay Function Analysis

Valid intra-chromosomal contact pairs were binned in 100 bp intervals, and the contact frequency for each bin was calculated by dividing to the total read count. We sorted contact pairs by orientations noted as “in-in (+/-),” “in-out (+/+),” “out-in (-/-),” and “out-out (-/+)”. Smoothed decay curves for each orientation were generated by using the CubicSpline function in the scipy.interpolate package from Python.

Interaction Hotspot Analysis

According to the decay function analysis (**Fig S13b**), the majority of contacts from Tri-HiC were short range (< 1 kb) in-in self-ligation products, which can be considered as a roughly even background over the genome. Taking the advantage of this property, we defined the interaction hotspots as regions having a significantly higher fraction of long range contacts, with the short range and long range being respectively defined as smaller and greater than 1.5 kb. To identify these hotspots, bam alignments obtained from the Distiller pipeline were split to short- and long-range interaction tracks based on the interaction distances recorded in the .pairs file. Reads were piled up and converted to coverage signals in .bedGraph format, using a binning window of 500 bp in 100 bp sliding step. For each bin, the expected long range read count was calculated by multiplying the short-range count of the bin by the average long-range to short-range ratio over the 20 kb neighboring background.. The statistical significance for the difference between expected and actual long range contacts was determined by Poisson statistics using the MACS2 bdgcmp –m ppois function[18]. Significant bins with p values smaller than the Bonferroni threshold (3E-7) were merged, and intervals with at least 300 bp span were considered interaction hotspots.

The -log10(p) score from the analysis corresponded to the distal interactivity track displayed in **Fig 4a**, and the average bin score for each peak was reported as its interactivity score for **Fig 4c**.

Loop Calling and Strength Analysis

Chromatin interaction loops were called by using the HiCCUPS algorithm from Juicebox[27]. We used the CPU version of HiCCUPS which, as described by the developer, searched for intrachromosomal loops within 8Mb of the diagonal. Loops were called with the following parameters: -k KR -r 200,500,1000,2000,5000,10000 -f 0.1 -p 5,4,2,2,2,2 -i 10,8,4,4,4,4 -t 0.02,1.5,1.75,2 -d 2000,4000,4000,8000,20000,20000. The same parameters were applied to the public *in situ* HiC results for IMR-90 at 5 kb and 10 kb resolutions.

The HiCCUPS reported both observed read count in the loop regions as well as the expected values from the donut background (expectedDonut), and their ratio defined the signal-to-background fold change of the loop, which we referred to as the loop strengths. Similarly, horizontal and vertical stripe strengths were calculated by dividing the expectedH and expectedV columns, respectively, to the expectedDonut column. The residual loop strength used for motif analysis was defined by the subtraction of log2 horizontal and vertical stripe strengths from log2 loop strength.

The intensities of significant loops identified by HiCCUPS were assessed by aggregate peak analysis (APA) also available from Juicebox with the following parameters: -n 500 -w 125 -r 200 -q 80 -k KR. APA scores from the analysis were directly reported in **Fig 3d**. The intensity matrices obtained from the analysis were transferred to Z-scores and presented in **Fig 3d**. The raw APA matrices were also used to evaluate the relative intensities of corners, stripes, and loops for the insulation analysis in **Fig S18d** by calculating the log 2 mean intensity of each interval indicated in the figure and normalizing against the left-bottom corner.

For the pile-up study at the vicinity of interaction hotspots, the APA was applied with the following parameters: -n 0 -w 250 -r 100 -q 320 -k KR. A distance-based normalization was applied to the obtained matrix by dividing each diagonal to the corresponding diagonal mean from a larger APA obtained with the parameters -n 0 -w 2500 -r 100 -k KR. The resulting matrix was Z-score transformed and shown in **Fig S18a**.

Motif Analysis

To annotate the interaction hotspots and loop anchors identified by Tri-HiC, we used the AME[43] package in the MEME suite to predict motifs in the intervals, with the accession to the JASPAR2018 and TFBSshape databases and default threshold parameters.

Insulation Analysis

Genome-wide insulation scores were calculated at 200 bp resolution by using the diamond-insulation function in the cooltools[44] package with the default parameters. In **Fig S18b**, we piled up the scores within the 20 kb region of all interaction hotspots categorized by their annotations. Insulation strengths for each hotspot in **Fig S18c** represented the range (maximum-minimum) of the insulation score of the interval.

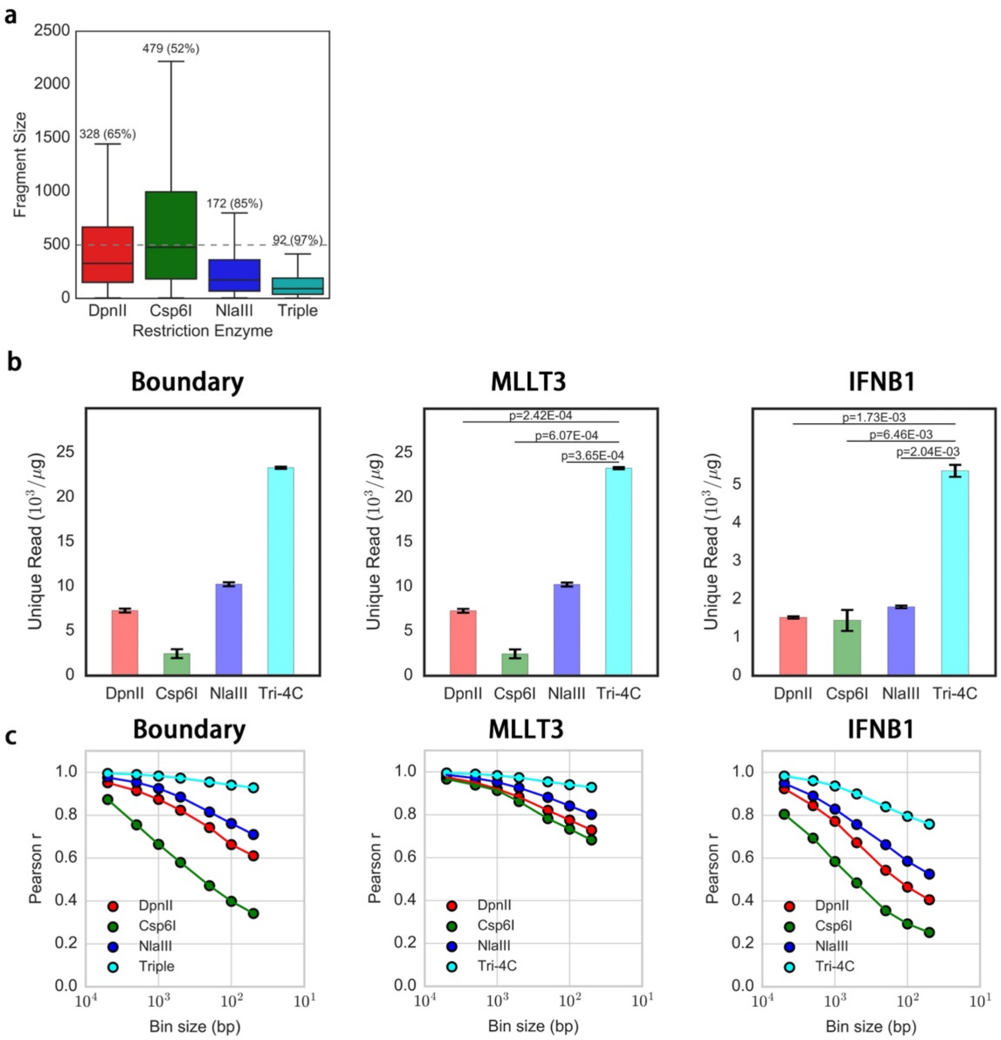

Figure S1 Tri-4C improves both yield and reproducibility by finer digestion of the genome. (**a**) Distribution of DNA fragment size of human genome digested by indicated restriction enzymes. Numbers on top indicate median and in the parentheses indicate percentage of fragments smaller than 500 bp window size. (**b**) Yield of unique intrachromosomal reads for UMI-4C and Tri-4C on the three viewpoints. (**c**) Reproducibility of interaction profiles binned in 50 kb-50 bp.

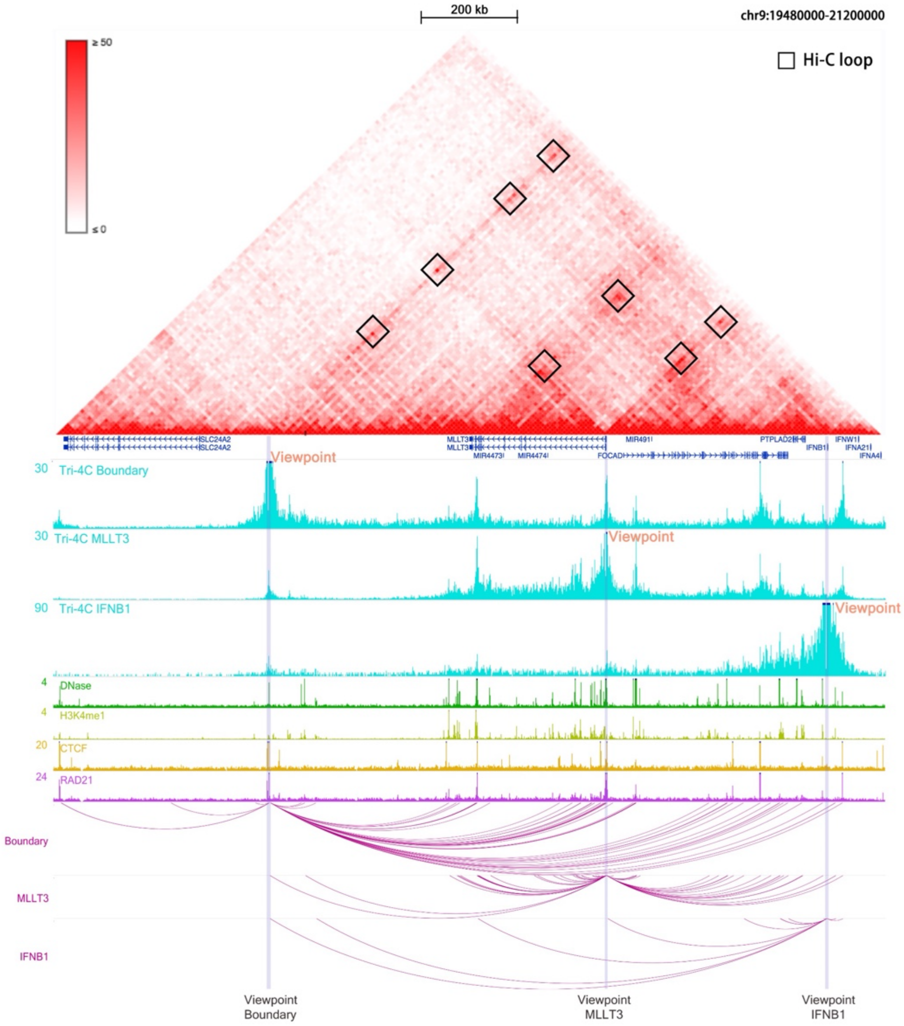

Figure S2 Overview of Tri-4C experimental design at the chromosome 9p21 *IFNB1* TAD. Tri-4C profiles of three viewpoints (Boundary, *MLLT3*, and *IFNB1*) are displayed under IMR90 *in situ* Hi-C matrix (5kb resolution Rao 2014) obtained from 3D genome browser (Hi-C loops are highlighted with squares). The Y axis of Tri-4C tracks denotes interaction frequency multiplied by 10,000. The interaction profiles are aligned with regulatory marks (DNase, H3K4me1) and boundary markers (CTCF, RAD21) for IMR90 cells obtained by the Roadmap Project. Bottom panel shows significant loop interactions between the viewpoints and CREs.

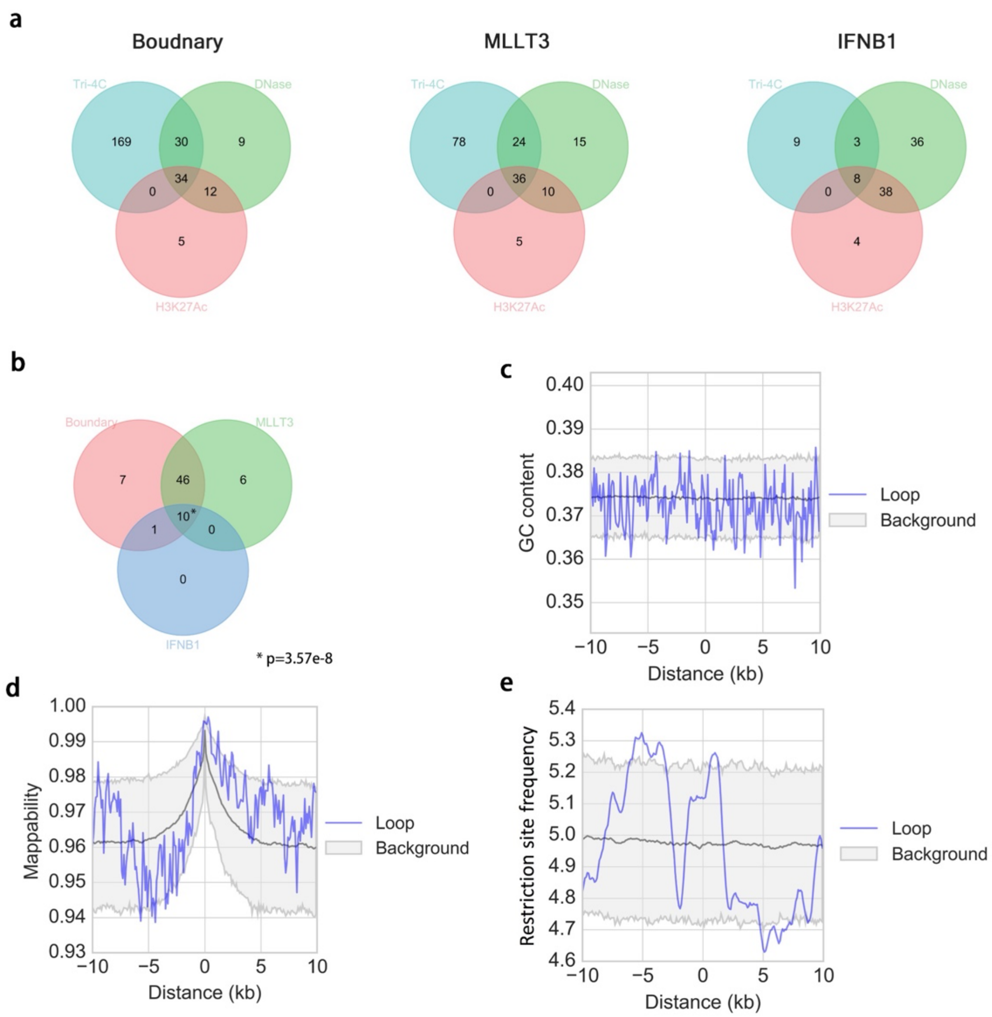

Figure S3 Tri-4C loop annotation and quality control. (**a**) Overlap between intra-TAD Tri-4C loops and intra-TAD DHS and H3K27ac peaks. (**b**) Overlap of DHS-marked CRLs among the three viewpoints. (**c**) GC content, (**d**) mappability, and (e) restriction site density around regions looped with any of the three viewpoints. Gray background indicates confidence intervals estimated by using 1,000 randomly selected intra-TAD regions not looped with any viewpoints with mappability > 0.5.

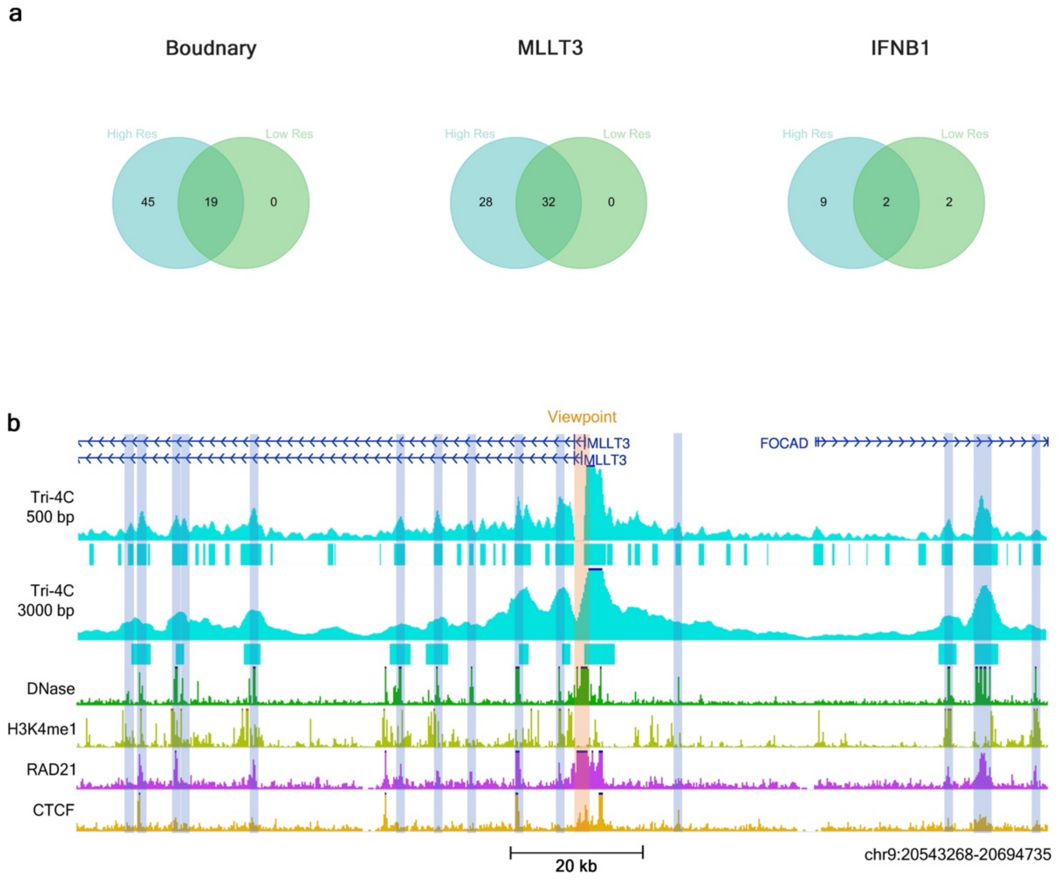

Figure S4 Comparison of Tri-4C profiles analyzed in 500 bp (High res) and 3000 bp (Low res) resolution. (**a**) Overlap of loops falling in CREs. (**b**) Interaction of *MLLT3* with neighboring CREs shown by Tri-4C in two resolutions. DHS peaks showing looping at 500 bp resolution are highlighted.

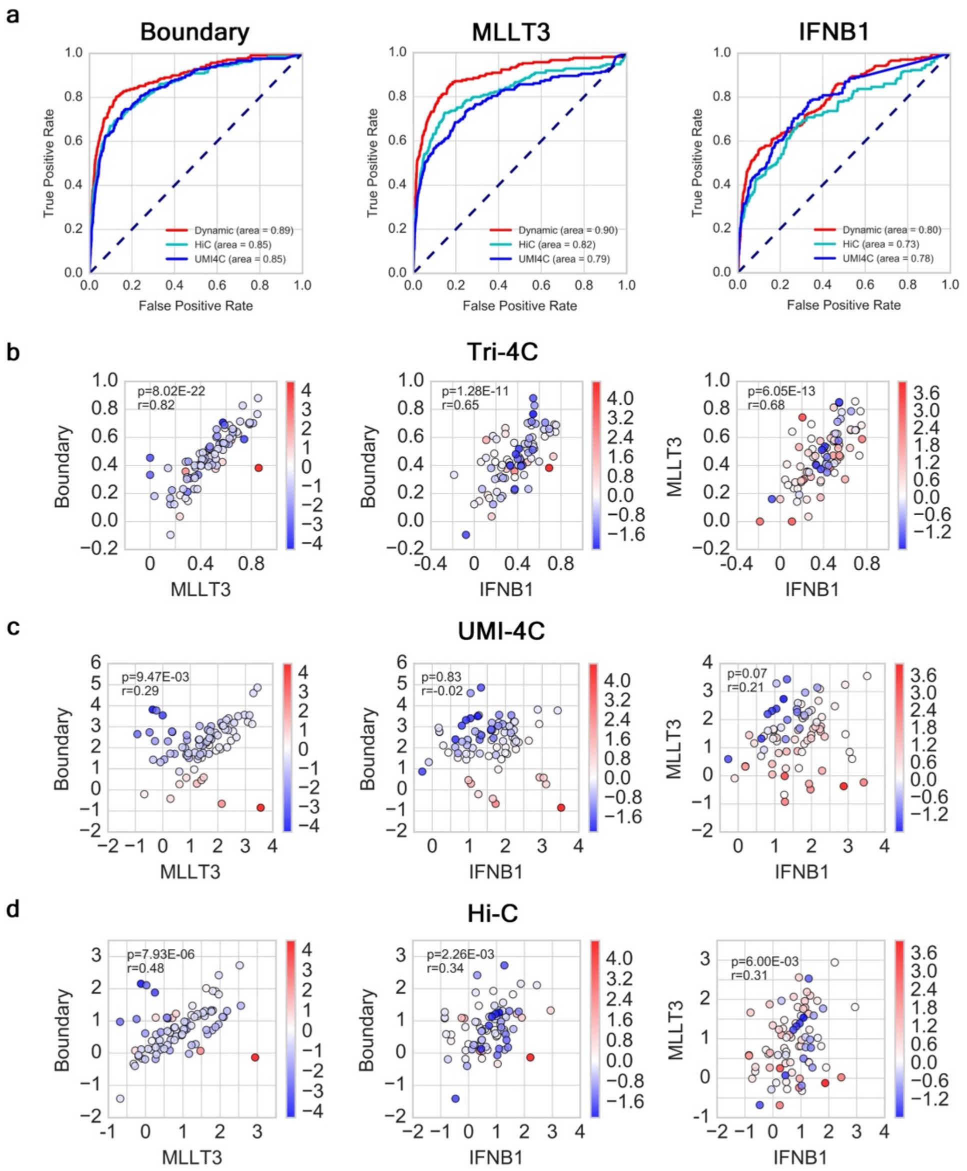

Figure S5 Comparison of loop calling algorithms. (**a**) ROC analysis using loop scores for each 100 bp bin steps calculated by Tri-4C (Dynamic), 1D Hi-C, and UMi-4C algorithms as predictors of intra-TAD DHS peaks. (**b**) 2D plots comparing loop strength (logFE) on all intra-TAD CREs determined by Tri-4C normalization, or read count using (**b**) UMI-4C or (**c**) Hi-C normalization between viewpoints. Color indicates log distance ratio between the x and y viewpoint (blue = closer to x and red = closer to y). Pearson correlation coefficient r and p value from linear regression are indicated.

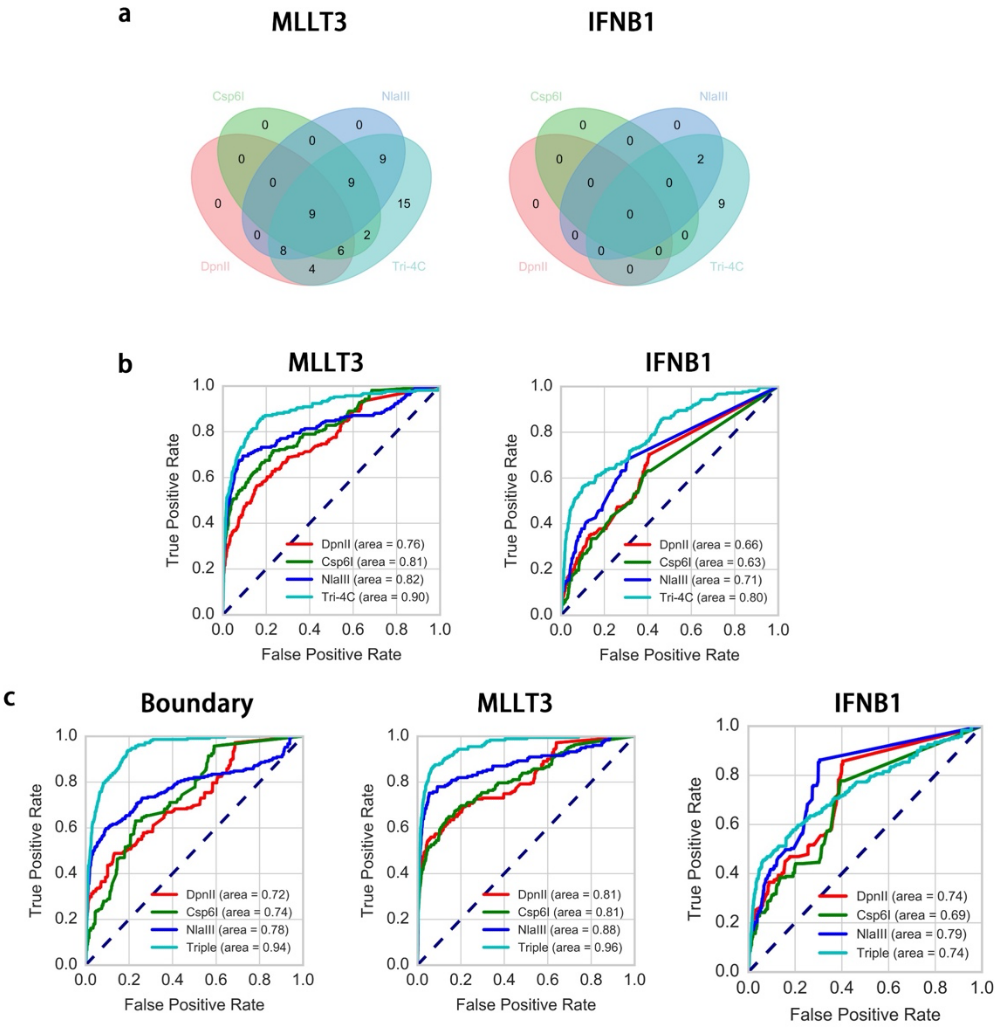

Figure S6 (**a**) Venn diagram of reproducible CRLs (N=2) called for *MLLT3* and *IFNB1* using Tri-4C and UMI-4C digested by three different restriction enzymes (**b**) ROC analysis using loop scores for each 100 bp bin as predictors of intra-TAD DHS peaks. (**c**) ROC analysis using loop scores for each 100 bp bin as predictors of intra-TAD H3K27Ac peaks.

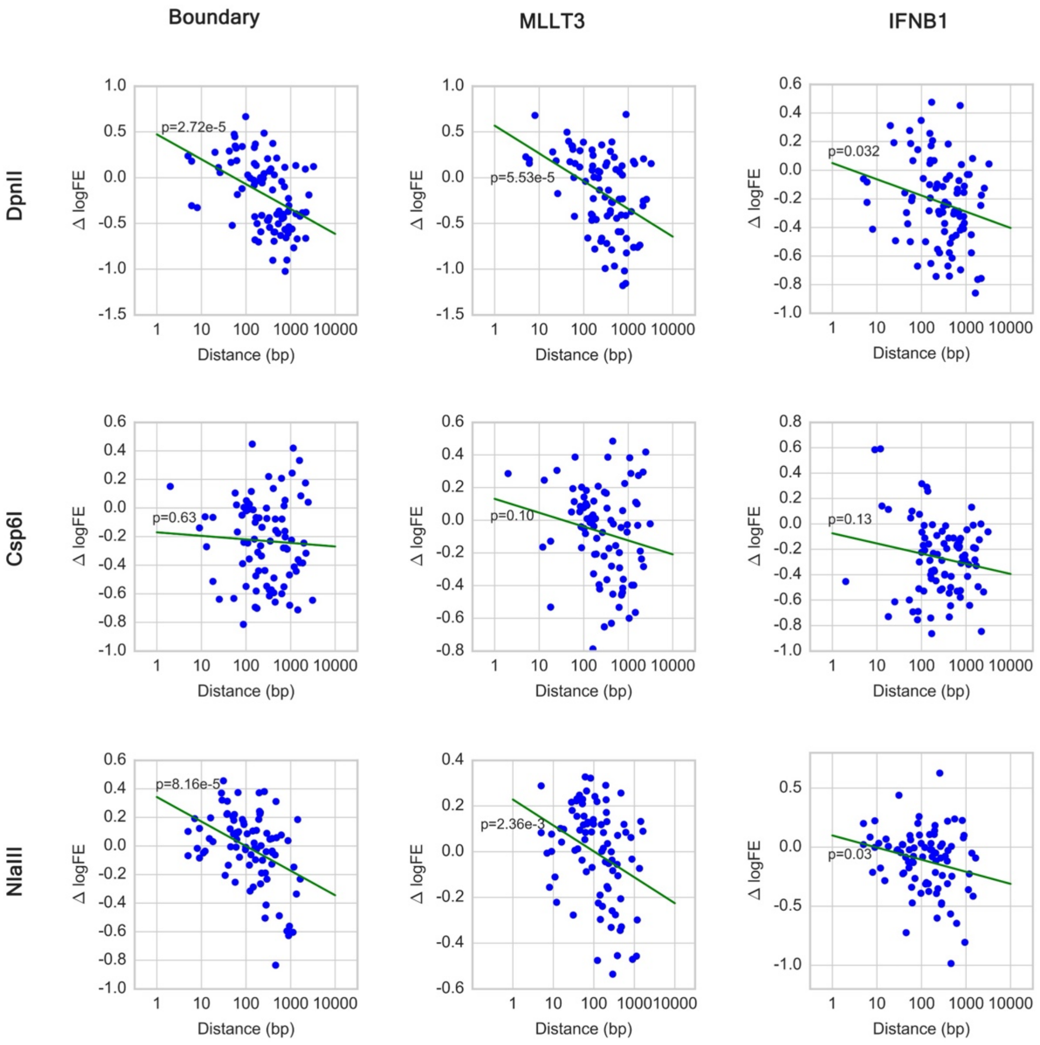

Figure S7 2D plots the loop strength difference between UMI-4C and Tri-4C (Y axis, ΔlogFE) and the distance between the nearest restriction site and the DHS peak center (X axis, log scale) of all intra-TAD CREs. The p values were calculated by fitting with linear regression.

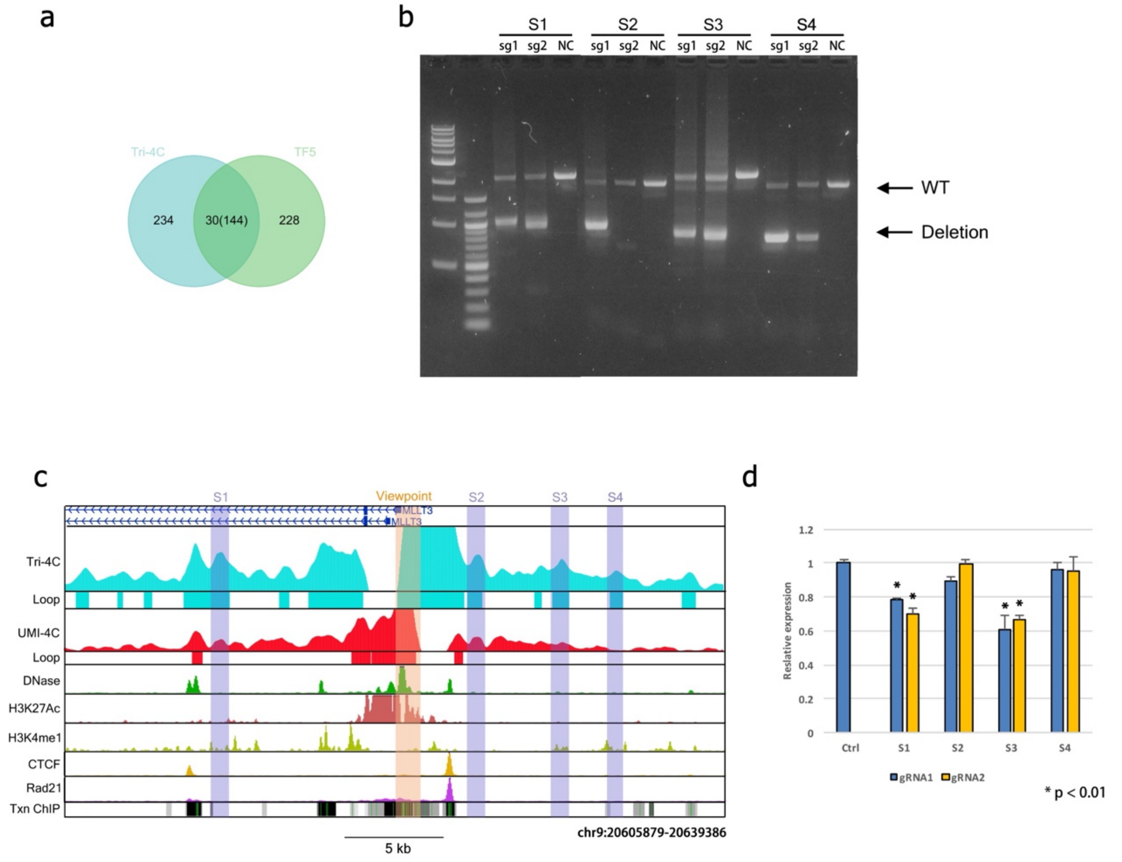

Figure S8 Analysis of Tri-4C loops that do not overlapped with CREs (**a**) Overlap between off-CRE loops within intra-TAD regions and ChIP-seq peaks of 5 or more transcription factor binding tracks combined in the ENCODE (Transcription Factor ChIP V3, 161 factors, all cell lines combined). (**b**) Validation of Cas9 deletion of S1-S4 indicated in (c). (**c**) Tri-4C, but not DpnII-UMI-4C, indicates looping of *MLLT3* with 4 neighboring regions (S1-S4) lacking enhancer marks and CTCF/cohesin. (**d**) Expression of *MLLT3* after deletion of these regions using Cas9 and two pairs of guide RNAs (sg1, sg2) quantified by real-time PCR (N=3).

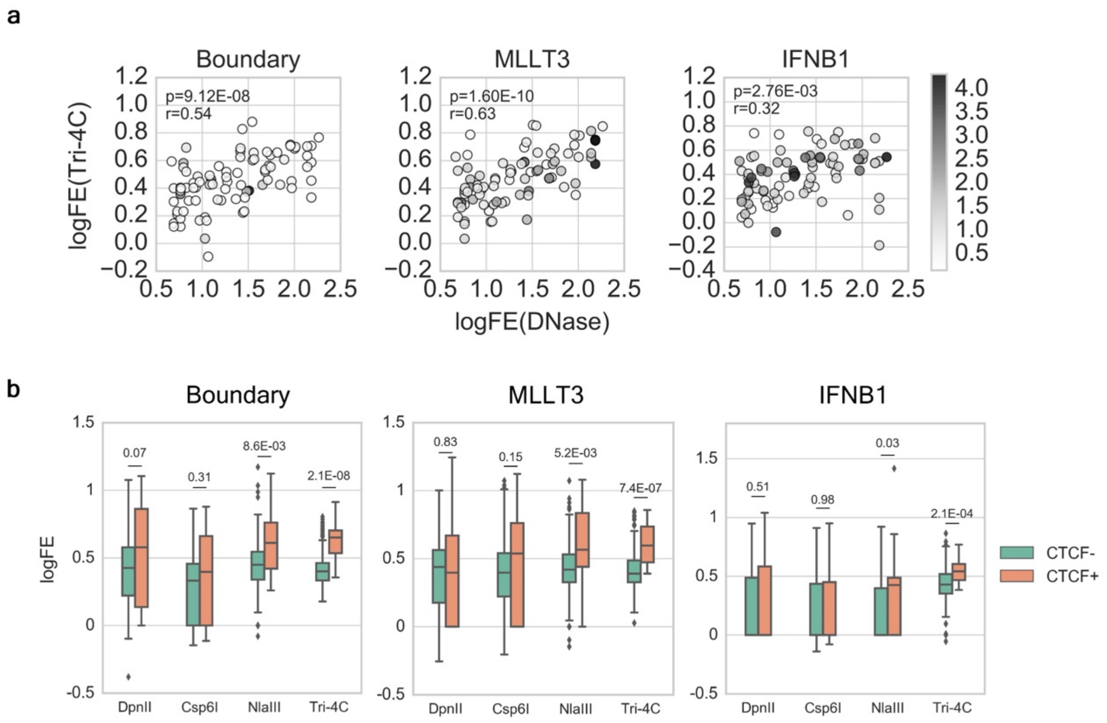

Figure S9 Analysis of Tri-4C loop strength. (**a**) Comparison between loop strength (logFE) from three viewpoints and DHS peak log fold enrichment on all intra-TAD CREs. Pearson correlation coefficient r and p value from linear regression model are indicated. (**b**) Association between loop strength and CTCF motif presence for Tri-4C and UMI-4C based on three enzyme digestion.

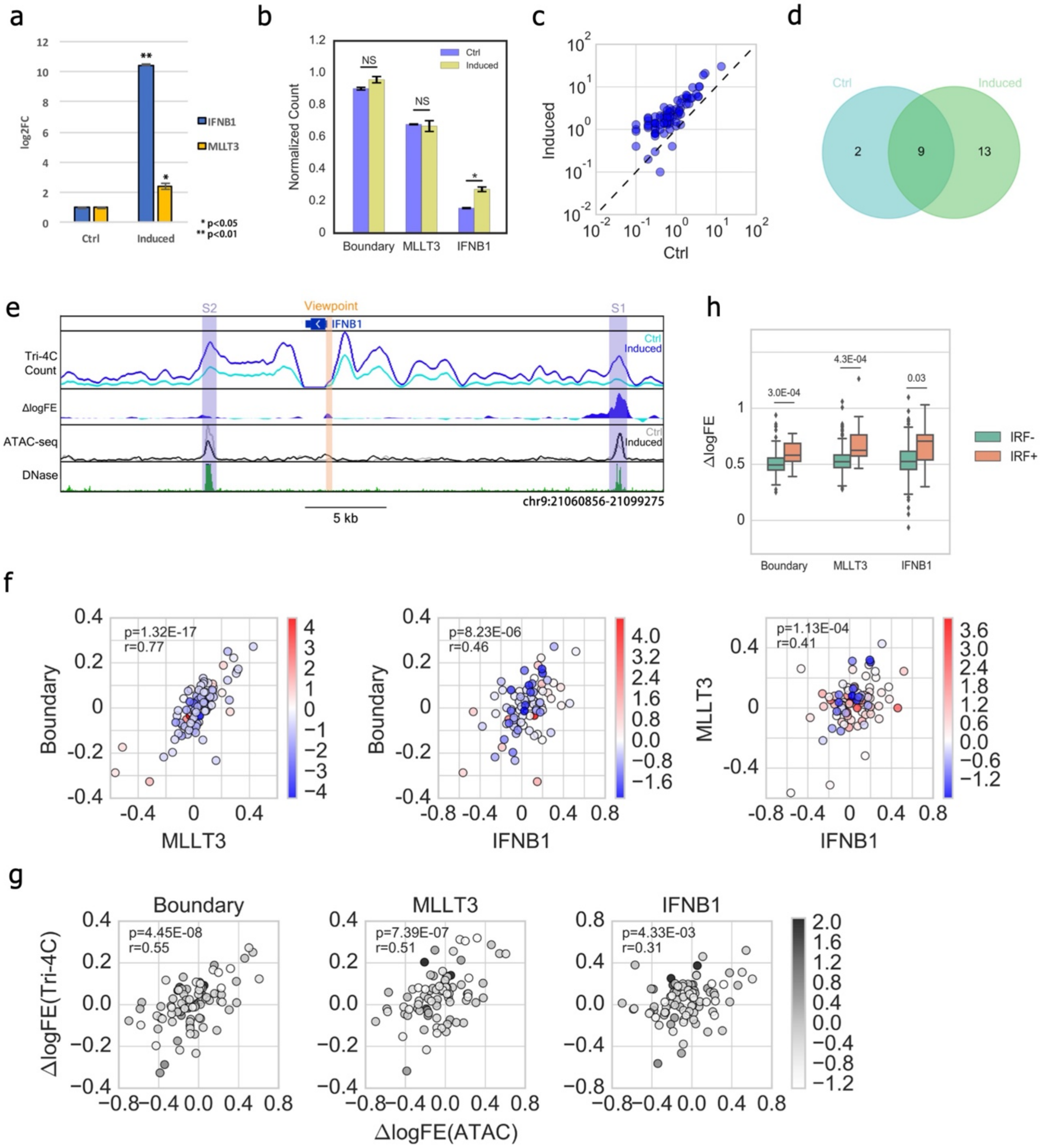

Figure S10 CRL alterations after *IFNB1* induction. (**a**) Expression profiling of *IFNB1* and *MLLT3* expression before and after *IFNB1* induction by using real-time PCR (N=3, t test). (**b**) Comparison of Tri-4C yield for three viewpoints (N=2) (**c**) 2D plot showing read count of *IFNB1* Tri-4C (normalized against Boundary) at all intra-TAD CREs before (X axis) and after (Y axis) induction. (**d**) Venn diagram showing the overlap of CRLs called from *IFNB1* before and after induction. (**e**) Alteration of *IFNB1* interaction before (Ctrl) and after (Induced) induced expression in IMR90. Top track aligns interaction read count, while second denotes loop strength alteration (ΔlogFE) and shows the loop gain is specific to S1 despite increased count on both S1 and S2. Third track indicates ATAC-seq peak signal of the two enhancers corresponding to the two conditions. S1 is a known enhancer of *IFNB1*[45]. (**f**) Comparisons of loop strength alterations (ΔlogFE) between three viewpoints and (**g**) with ATAC-seq peak log fold enrichment changes on all intra-TAD CREs. Pearson correlation coefficient r and p value from linear regression model are indicated. (**h**)

Association between loop strength alterations after *IFNB1* induction and IRF(1/2/3/7) motif presence at intra-TAD CREs. Statistical p values were calculated by the U test.

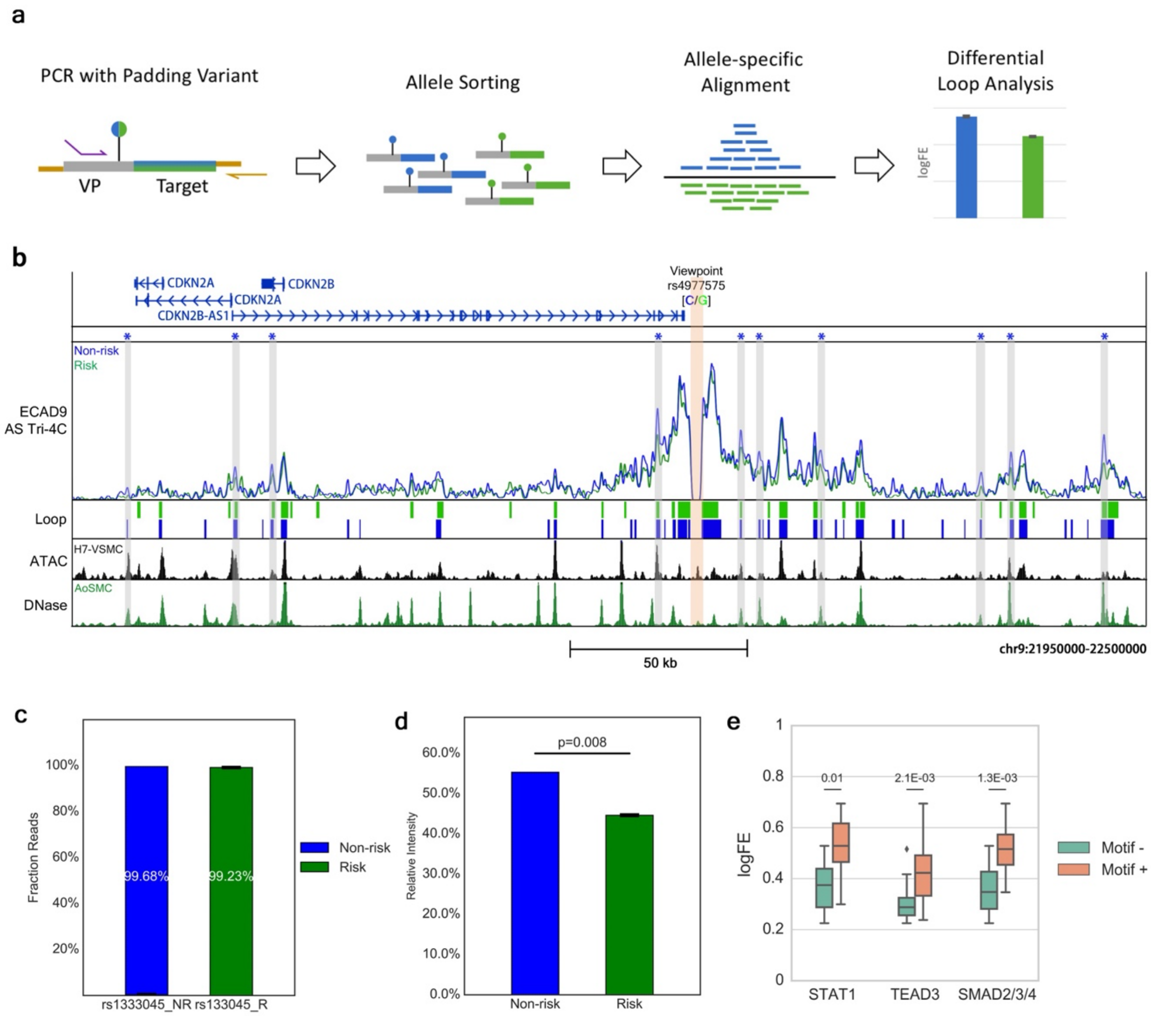

Figure S11 Allele-specific Tri-4C for ECAD9. (**a**) Schematics for the allele-specific study design. The viewpoint primer is designed to include a heterozygote flag variant in the padding sequence. Reads are sorted and mapped separately according to the variant genotype. Allele-specific interaction loops are identified by differential loop analysis. (**b**) Allele-specific (AS) Tri-4C profile for ECAD9 in H7-derived VSMCs. Intervals below indicate loop regions called by each allele. Loops on ATAC-seq-marked CREs showing significant allelic bias (FDR < 0.05, Methods) are highlighted with * marks, with its color indicating the stronger allele. The VSMC ATAC-seq and ENCODE aortic smooth muscle cell (AoSMC) DNase tracks are shown below. (**c**) Fraction of *cis* interaction mapped to each AS profile (N=2) (**d**) ATAC-dPCR (digital PCR) on ECAD9. Significant p value was calculated by t test (N=2). **(e)** Distribution of loop peak strengths with target DNase peaks with or without indicate TF motifs.

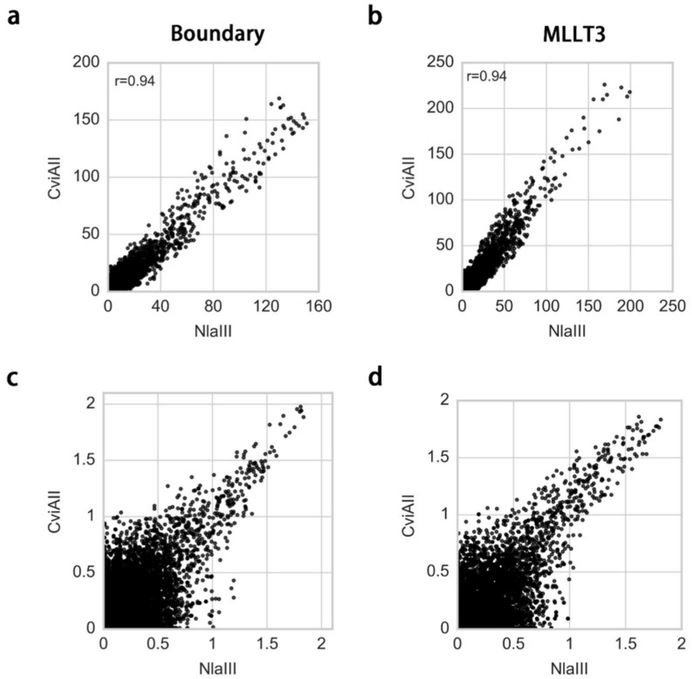

Figure S12 Reproduction of Tri-4C using alternative digestion by CviAII. (**a**) 2D plot showing read count in 500 bp bins obtained from original Tri-4C and alternative digestion protocol. Pearson correlation coefficient r is indicated. (**b**) Loop score (log(-log(p)) comparison for all bins scored above 0 (p<0.1).

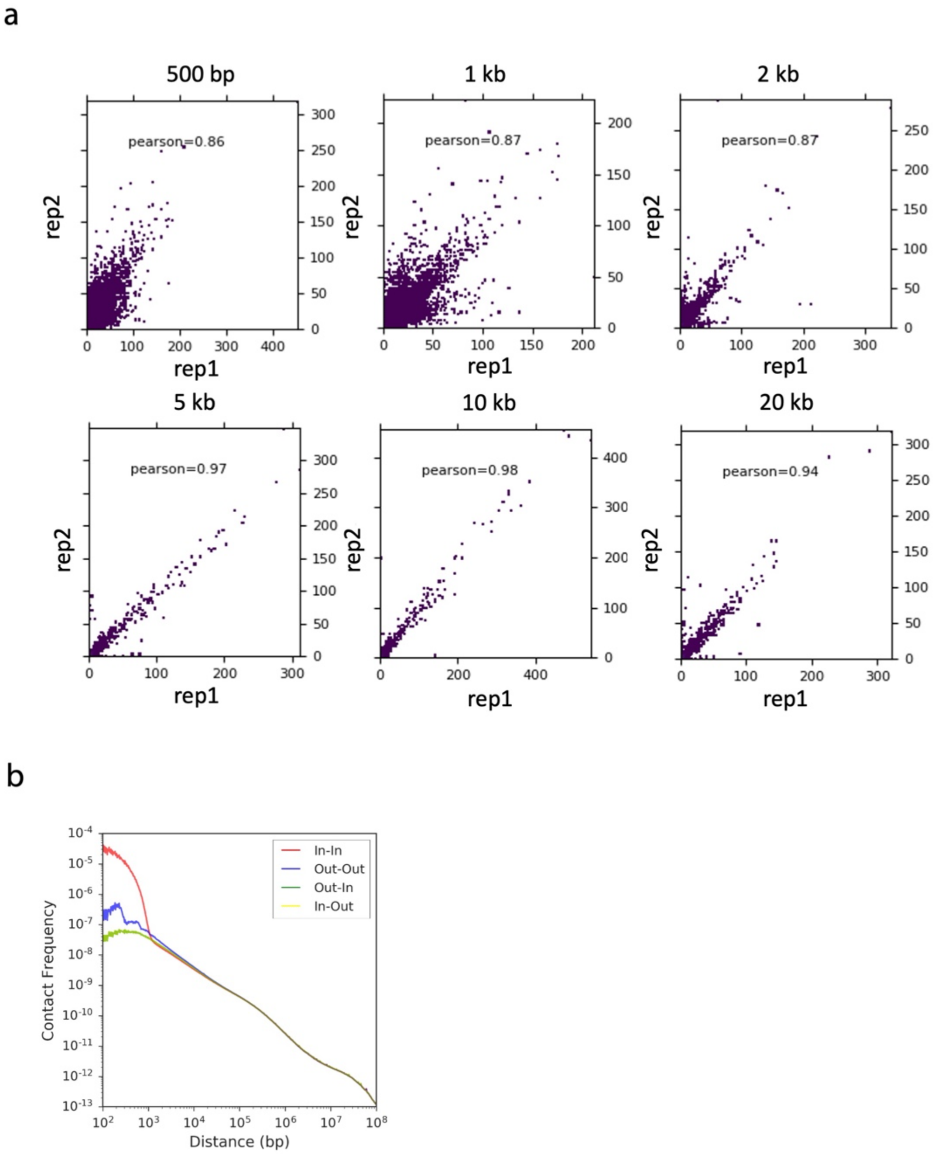

Figure S13 (**a**) Reproducibility of Tri-HiC at various resolutions for intrachromosomal contacts within 8 Mbp range (same as the distance restraint for loop calling). (**b**) Contact frequencies versus distance for read pairs in indicated directions determined by Tri-HiC.

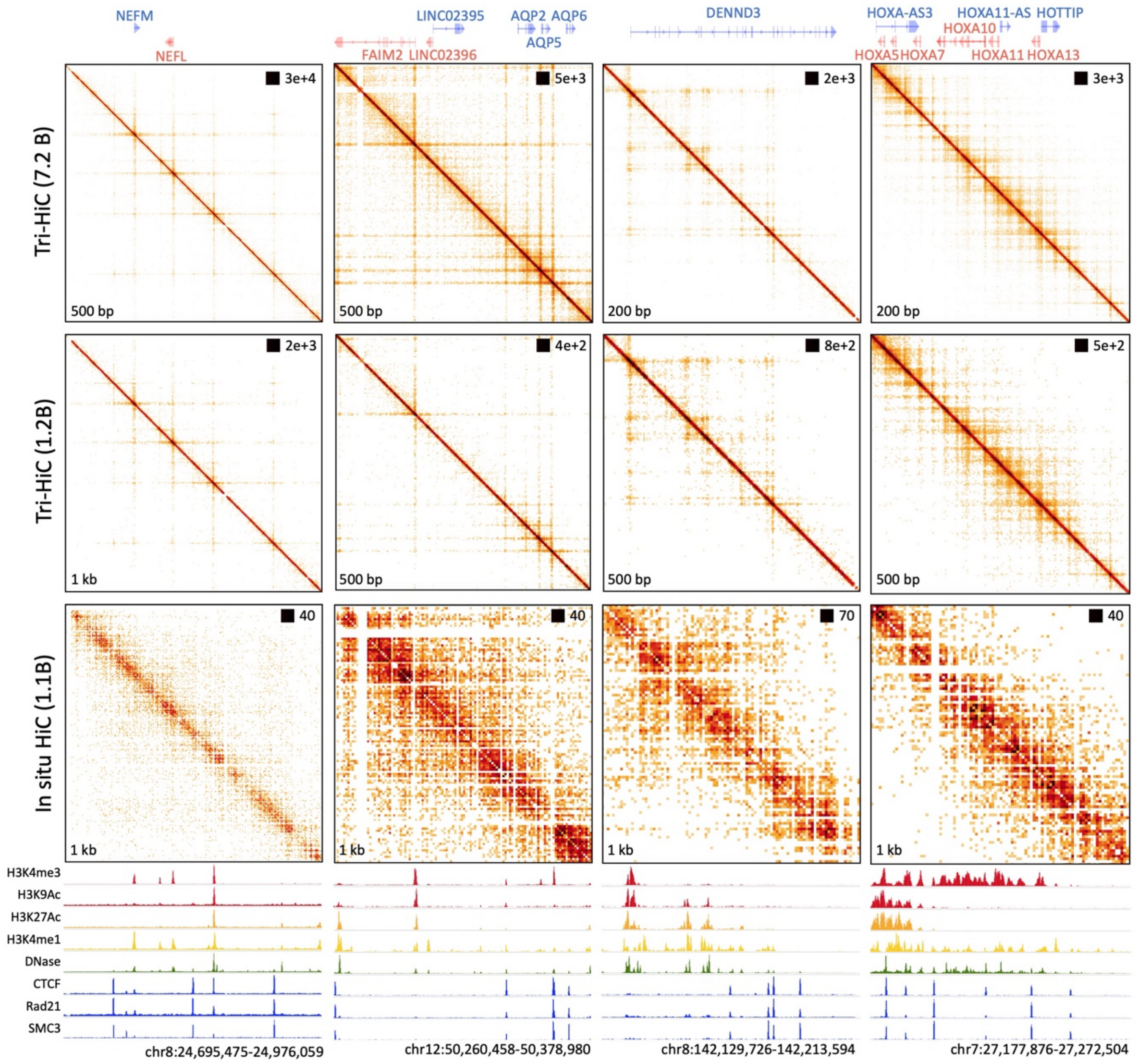

Figure S14 Comparisons of IMR90 interaction maps between Tri-HiC (all replicates, 7.2 billion contacts), Tri-HiC (replicate #2, 1.2 billion contacts), and *in situ* HiC in at sub-kilobase resolutions in 4 chromosomal loci.

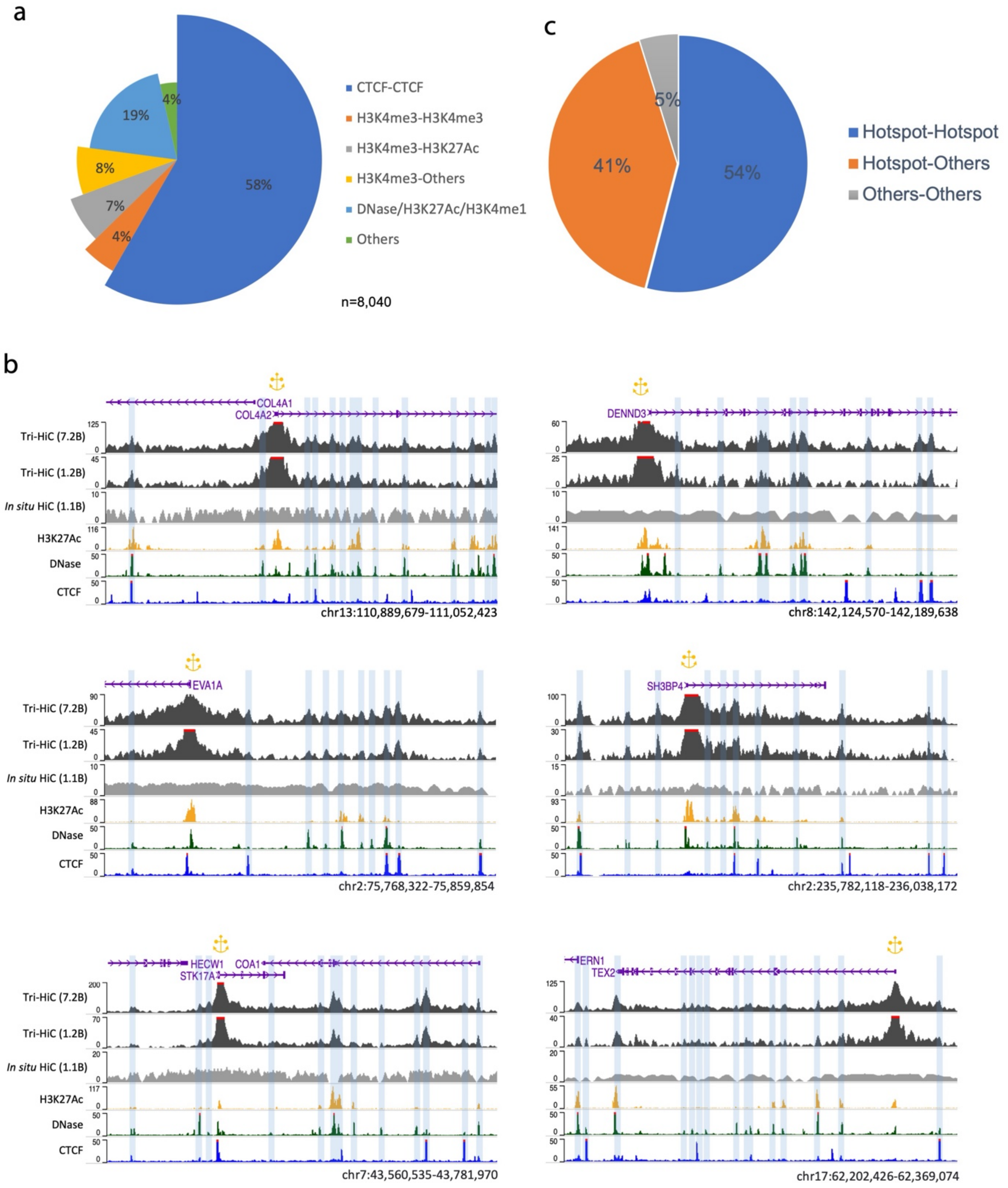

Figure S15 (**a**) Annotation of *in situ* HiC loops. (**b**) Examples of virtual 4C derived from Tri-HiC for promoters interacting with multiple enhancers Additional annotations of Tri-HiC-identified loops. (**c**) Annotation of 1 kb Tri-HiC loops.

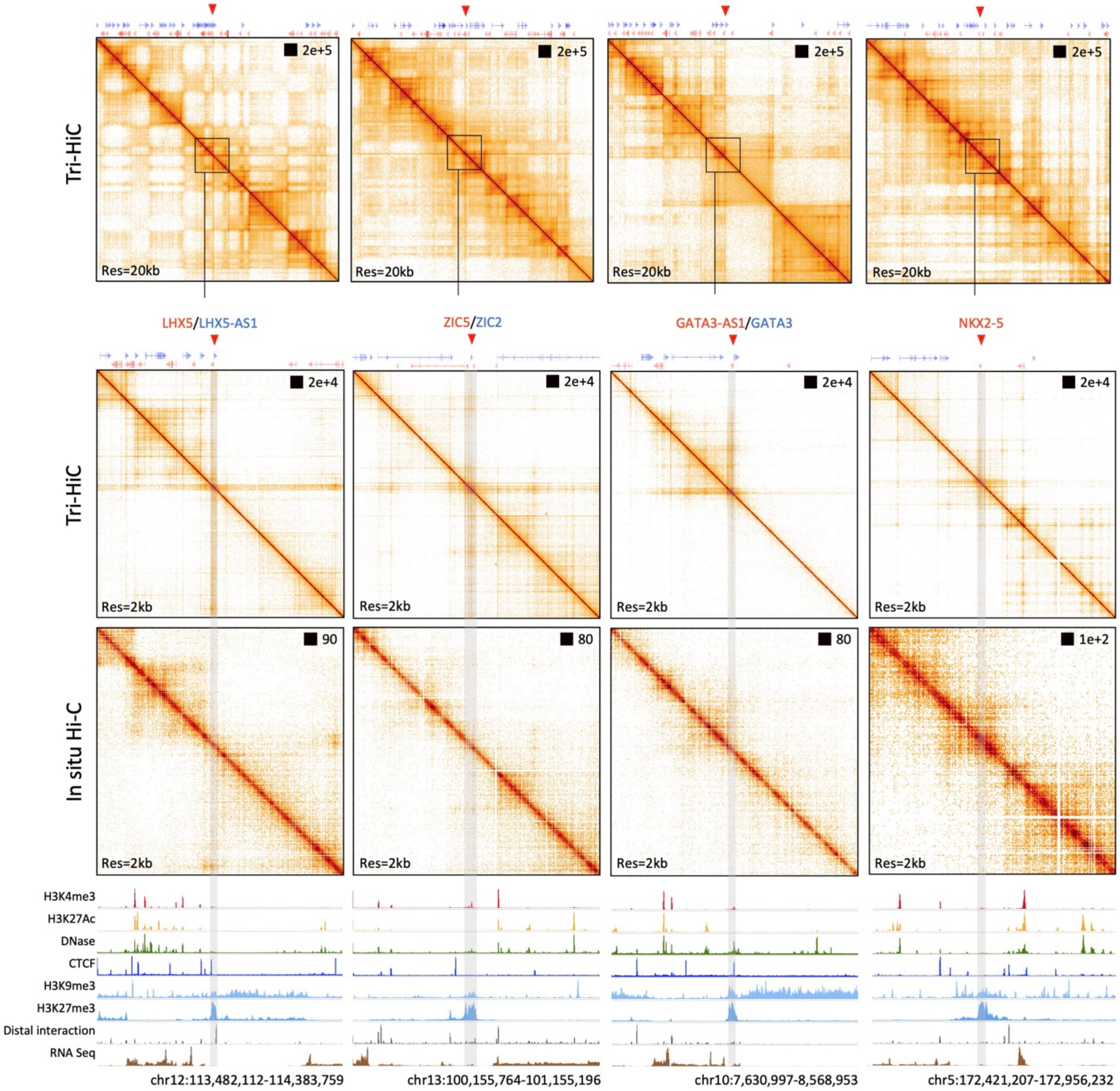

Figure S16 Examples of H3K27me3-associated super-stripes (highlighted by gray bars) identified by Tri-HiC.

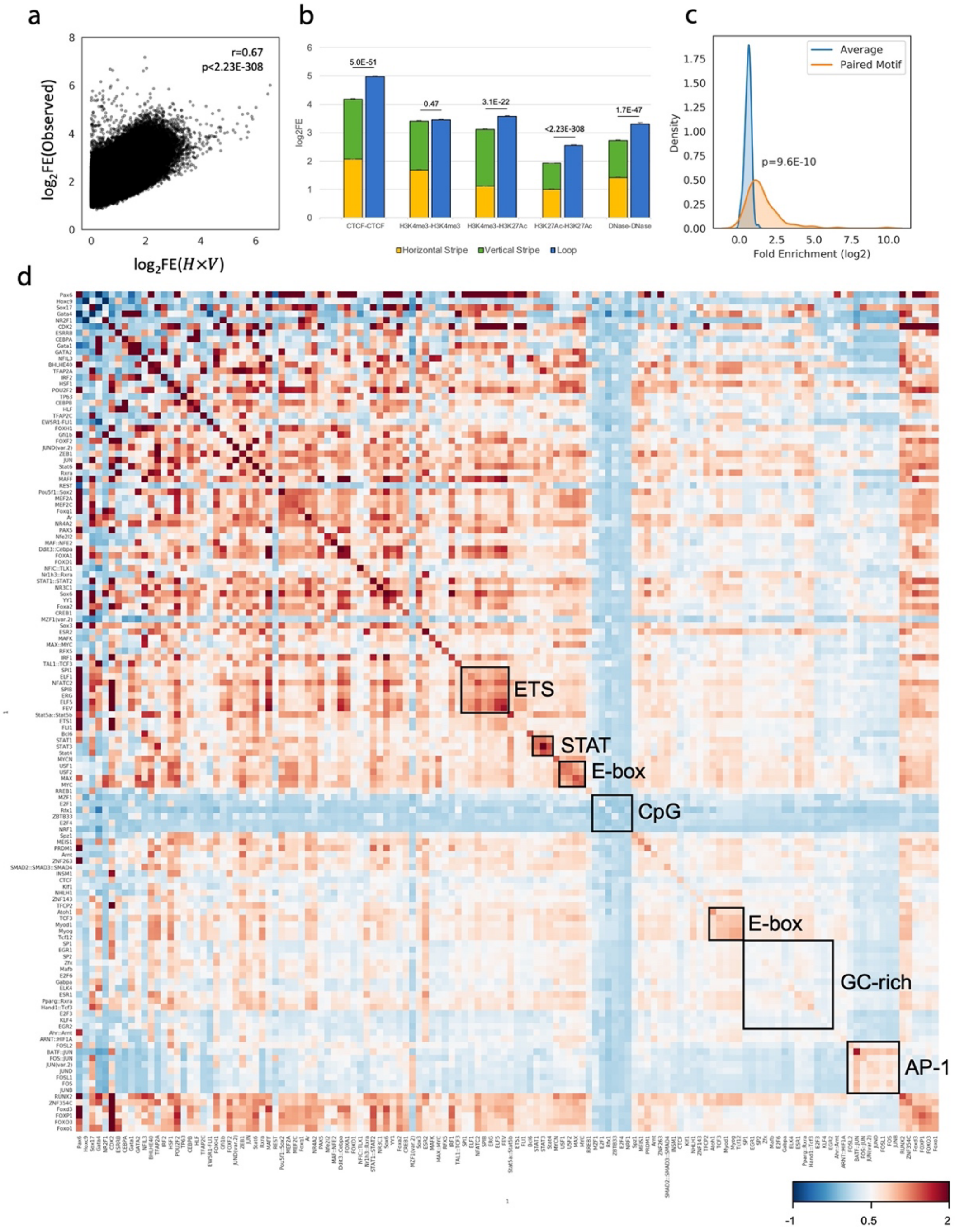

Figure S17 Decomposition analysis of Tri-HiC loops. (**a**) Scatter plot showing correlations between loop fold enrichment (FE) and product of stripe fold enrichments of the two corresponding loop anchors around the loop sites. See Methods for precise definition of loop and stipe regions. (**b**) Categorical comparisons between loop and stripe fold enrichments. Loops annotated into multiple categories are only included in the leftmost category. Numbers above the

bars indicate p values for the differences determined by two-sample t tests. (c) Distributions of residual loop strengths for each TF identified in pairs on both loop anchors (i.e. diagonal of (d)), in comparison with the distribution of average residual loop strength for each TF (i.e. row average). Significance of differential distribution was calculated by paired t-test. (d) Heatmap of average residual loop strengths for all 1 kb loops identified by Tri-HiC with indicated TF motif pairings.

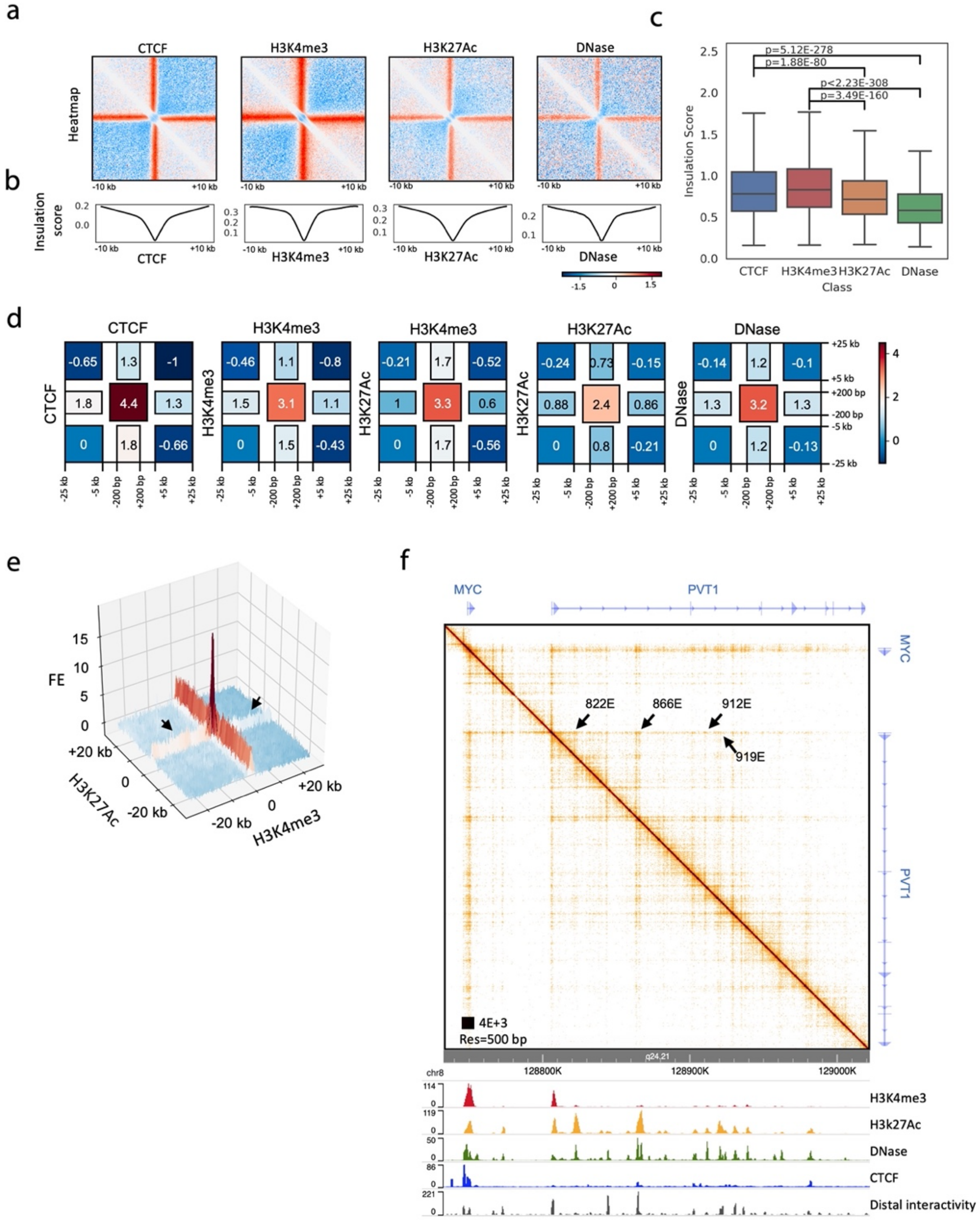

Figure S18 Tri-HiC reveals chromatin insulation associated with cis-regulatory elements. (**a**) Distance-normalized and Z score-transformed piled up heatmaps of distal interaction hotspots annotated with indicated epigenetic marks at 100 bp resolution. (**b**) Piled-up insulation scores for regions indicated in (**a**). Comparison of insulation strength of interaction hotspots, defined as the range of insulation scores within their 20 kb neighboring regions, annotated with indicated marks.

Regions annotated into multiple categories are included only once into the leftmost category. (**d**) Relative log2 interaction intensities of the four corners of interaction loops with indicated annotations. (**e**) A 3D pile-up showing the asymmetry of promoter-active enhancer loops. Black arrows indicate reduction of enhancer (marked by H3K27Ac) stripe strengths after encountering the looped promoter (marked by H3K4me3). (**f**) Tri-HiC interaction heatmap of MYC-PVT1 locus in IMR90. Arrows indicate diminished interaction stripes of 4 enhancers upon interacting with the PVT1 promoter.

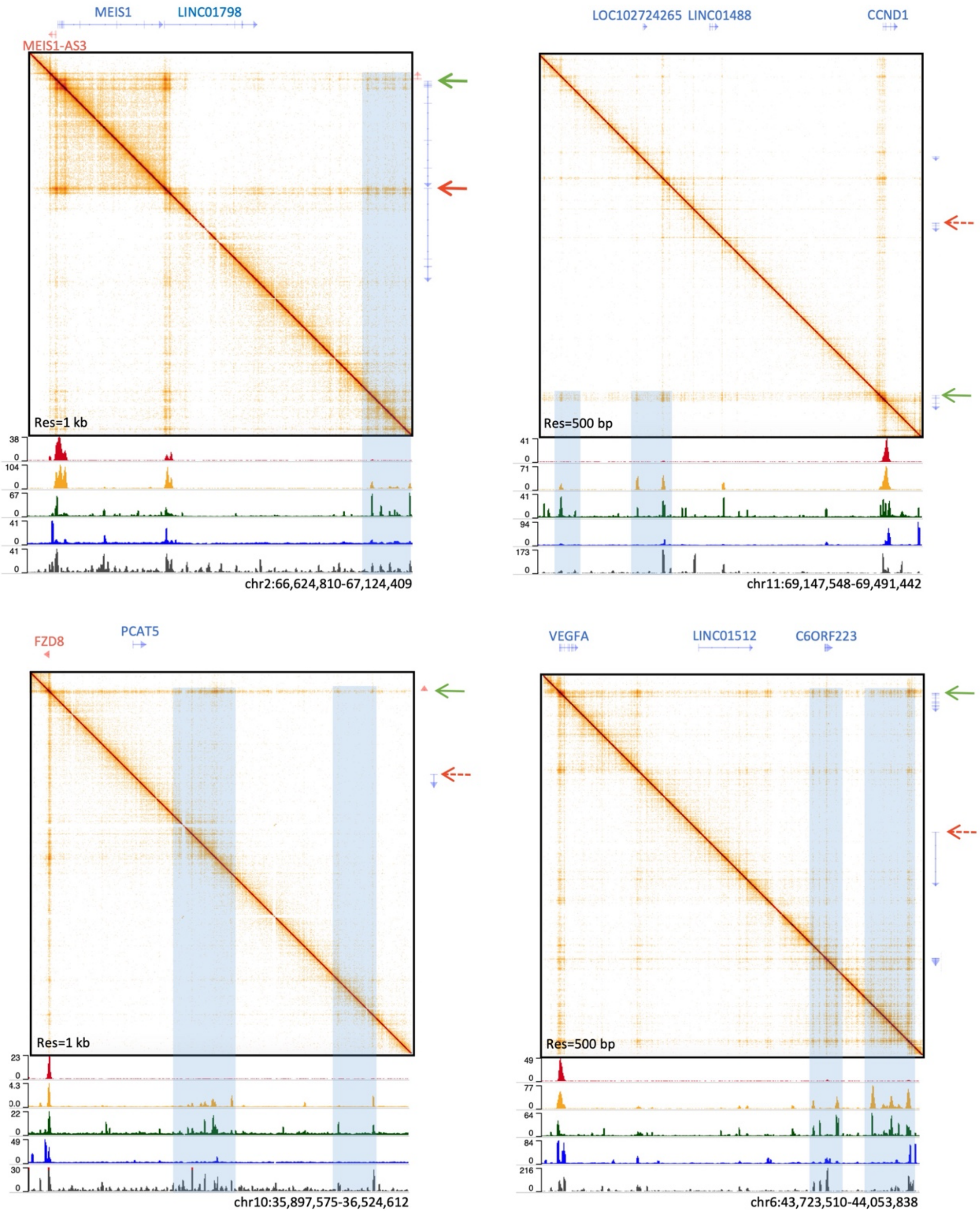

Fig S19 Examples of loci where lncRNAs are located between the oncogene and its looped enhancer networks. For MEIS1 (green arrow), interactions with the downstream enhancers is interfered with by *LINC01798*(red arrow) promoter, whereas for CCND1, FZD8, and VEGFA, the promoters of respective lncRNAs (*LINC01488, PCAT5*, and *LINC01512*, dotted red arrow) are not occupied with active histone markers and show no sign of interaction insulation.

Reference

39 Tan, G. & Lenhard, B. TFBSTools: an R/bioconductor package for transcription factor binding site analysis. Bioinformatics 32, 1555-1556, doi:10.1093/bioinformatics/btw024 (2016).

40 Khan, A. et al. JASPAR 2018: update of the open-access database of transcription factor binding profiles and its web framework. Nucleic Acids Res 46, D260-D266, doi:10.1093/nar/gkx1126 (2018).

41 Kerpedjiev, P. et al. HiGlass: web-based visual exploration and analysis of genome interaction maps. Genome Biol 19, 125, doi:10.1186/s13059-018-1486-1 (2018).

42 Wolff, J. et al. Galaxy HiCExplorer: a web server for reproducible Hi-C data analysis, quality control and visualization. Nucleic Acids Res 46, W11-W16, doi:10.1093/nar/gky504 (2018).

43 McLeay, R. C. & Bailey, T. L. Motif Enrichment Analysis: a unified framework and an evaluation on ChIP data. BMC Bioinformatics 11, 165, doi:10.1186/1471-2105-11-165 (2010).

44 Abdennur, N. & Mirny, L. A. Cooler: scalable storage for Hi-C data and other genomically labeled arrays. Bioinformatics 36, 311-316, doi:10.1093/bioinformatics/btz540 (2020).

45 Banerjee, A. R., Kim, Y. J. & Kim, T. H. A novel virus-inducible enhancer of the interferon-beta gene with tightly linked promoter and enhancer activities. Nucleic Acids Res 42, 12537-12554, doi:10.1093/nar/gku1018 (2014).

Dataset S1 Sequences for viewpoint-specific primers.

Dataset S2 Statistics for Tri-4C and UMI-4C libraries. Total Read indicates actual sequencing depth. On Target Ratio indicates reads with matched padding sequence. Unique Read indicates yield after deduplication. Intra-TAD Ratio indicates reads falling into the same TAD as the viewpoint.

Dataset S3 Sequences for gRNA and validation primers used for *MLLT3* putative enhancer deletion.

Dataset S4 Summary Statistics for IMR90 Tri-HiC libraries.
